# CREM Marks TCR-Driven T-Cell Activation and Associates with Favorable Prognosis in Papillary Thyroid Carcinoma: A Single-Cell and Bulk Transcriptomic Study

**DOI:** 10.3390/ijms27167387

**Published:** 2026-08-18

**Authors:** Hao Ling, Peiyu Qiu, Yanzhu Hu, Yan Sun

**Affiliations:** 1 Department of Surgery, Klinikum Rechts der Isar, TUM School of Medicine and Health, Technical University of Munich, 81675 Munich, Germany; hao.ling@tum.de (H.L.); yanzhu.hu@tum.de (Y.H.); 2 Department of Respiratory Medicine, Institute for Nutrition and Translational Research in Metabolism (NUTRIM), Maastricht University Medical Center, 6229 HX Maastricht, The Netherlands; peiyu.qiu@maastrichtuniversity.nl; 3 Department of Genetics & Cell Biology, Institute of Nutrition and Translational Research in Metabolism (NUTRIM), Institute for Oncology and Reproduction (GROW), Maastricht University, 6229 ER Maastricht, The Netherlands

**Keywords:** cAMP-responsive element modulator (CREM), papillary thyroid carcinoma, T-cell activation, immediate early genes, NR4A, progression-free interval, ANXA1

## Abstract

Although papillary thyroid carcinoma (PTC) is widely considered an immunologically cold tumor, detectable T-cell infiltration can influence disease progression in selected patients. Previous bulk analyses have linked cAMP-responsive element modulator (CREM) expression to a favorable progression-free interval and an inverse correlation with regulatory T-cell infiltration but have not resolved the cellular origin or functional state. In this study, we integrate single-cell RNA-sequencing data from a 23-sample PTC atlas with bulk Cancer Genome Atlas Thyroid Carcinoma (TCGA–THCA) validation. We show that CREM marks a T-cell receptor (TCR)-driven immediate early gene activation state in tumor-infiltrating T cells rather than a cAMP program; this state is consistently and modestly enriched within the FOXP3^+^ regulatory T-cell compartment, challenging the interpretation that bulk inverse Treg–CREM correlations reflect Treg-intrinsic repression. CREM-high T cells upregulate ANXA1, and permutation-controlled analysis identifies a candidate paracrine axis from CD4/CD8 T cells to the epithelial epidermal growth factor receptor (EGFR). At the bulk level, CREM expression correlates with reduced proliferation programs and is associated with a prolonged progression-free interval (multivariate HR 0.56, 95% CI 0.31–1.00, *p* = 0.050), with the effect direction preserved after immune and sex adjustment. Genome-wide differential expression and gene-set enrichment in an independent cohort (GSE193581) confirm that CREM-detected T cells are enriched for a TCR-driven immediate early transcriptional program, supporting the reinterpretation of CREM as a marker of TCR-driven IEG activation.

## 1. Introduction

Papillary thyroid carcinoma (PTC) is the most common endocrine malignancy and behaves as an indolent solid tumor. For thyroid cancer, the 5-year overall survival rates reach approximately 98.5%, with excellent long-term outcomes documented in localized disease [1]. Despite this favorable natural history, a clinically important subset of patients develops distant metastases or radioiodine-refractory disease, for which systemic treatment options remain severely limited [1,2].

Although PTC is characterized as a relatively immunologically “cold” tumor, marked by a low tumor mutational burden, limited CD8^+^ T-cell infiltration, and a non-established role for immune checkpoint blockade (ICB) as a first-line standard of care [3,4], PTC tissues frequently harbor detectable T-cell populations alongside the overexpression of key immune checkpoints. Consequently, novel immune-based stratification approaches are actively being explored to optimize and guide ICB therapies [5,6]. These observations underscore the critical need for compartment-resolved mechanistic insight into T-cell functional states within the PTC tumor microenvironment (TME) that directly shape tumor behavior or therapeutic responsiveness.

As a pivotal cyclic AMP (cAMP)-responsive transcription factor, the cAMP-responsive element modulator (CREM) plays a foundational role in modulating T-cell responses by governing the transcription of multiple Effector cytokines, including IL-2, IL-17, and IL-21 [7]. Upon acute T-cell activation, CREM is transiently induced and serves as a transcriptomic rheostat, binding to the *IL2* promoter to suppress its transcription through chromatin-dependent mechanisms [8]. In chronic autoimmune conditions such as systemic lupus erythematosus (SLE), the specific CREMα isoform is stably overexpressed in T cells. This CREMα isoform directly drives a pathogenic Effector phenotype by epigenetically repressing *IL2* while activating *IL17A* and *IL21* [9,10,11]. However, although the regulatory contributions of CREM within autoimmunity are increasingly mapped, its specific functional programming, downstream paracrine outputs, and clinical relevance within the solid tumor immune landscape remain poorly characterized.

Our previous bulk-RNA-seq analysis of TCGA–THCA demonstrated that CREM is downregulated in tumor versus normal thyroid tissue; independently predicts longer progression-free intervals (PFIs); and inversely correlates with regulatory T-cell infiltration, Treg-recruiting chemokines, and multiple immune checkpoints [12]. The bulk measurements represent weighted averages across heterogeneous cell populations. Despite these findings, three fundamental questions remain unresolved: (i) which T-cell functional program(s) CREM marks at single-cell resolution: canonical cAMP signaling, TCR/NFκB-driven immediate early gene (IEG) activation, exhaustion, Effector cytotoxicity, or another state; (ii) whether the inverse bulk Treg–CREM correlation reflects Treg-intrinsic transcriptional repression or a compositional tradeoff in which highly activated T cells and Tregs are inversely abundant across samples; and (iii) whether candidate Nectin Cell Adhesion Molecule/Poliovirus Receptor (NECTIN/PVR) checkpoint axes identified in bulk data represent population-level signaling events under permutation-controlled cell–cell communication testing. Resolving the cellular origin and mechanistic outputs of the CREM signal is therefore essential for interpreting its prognostic value and evaluating its potential as a stratification biomarker. In this study, we addressed these gaps through single-cell RNA-sequencing (RNA-seq) analysis of a 23-sample PTC atlas (GSE184362) [13] integrated with bulk TCGA–THCA validation. We hypothesized that CREM marks a TCR/NFκB-driven IEG activation state driven by TCR/NFκB in tumor-infiltrating T cells rather than a generic cAMP program. Using Harmony-integrated single-cell profiling and a permutation-controlled CellChat inference, we demonstrate that CREM identifies an NFκB-driven IEG state paradoxically enriched in FOXP3^+^ regulatory T cells, propose a T-cell-secreted ANXA1 → epithelial epidermal growth factor receptor (EGFR) paracrine axis consistent with reduced tumor proliferation programs, and show that CREM independently predicts prolonged PFI after adjustment for clinical and immune-cell covariates. These findings reframe CREM as a compartment-resolved marker of T-cell engagement intensity and provide a mechanistic bridge between T-cell state and tumor behavior in patients with indolent thyroid cancer.

## 2. Results

### 2.1. A 23-Sample PTC Single-Cell Atlas Integrated by Harmony Reveals Ten Well-Resolved Lineages

**Figure 1:**
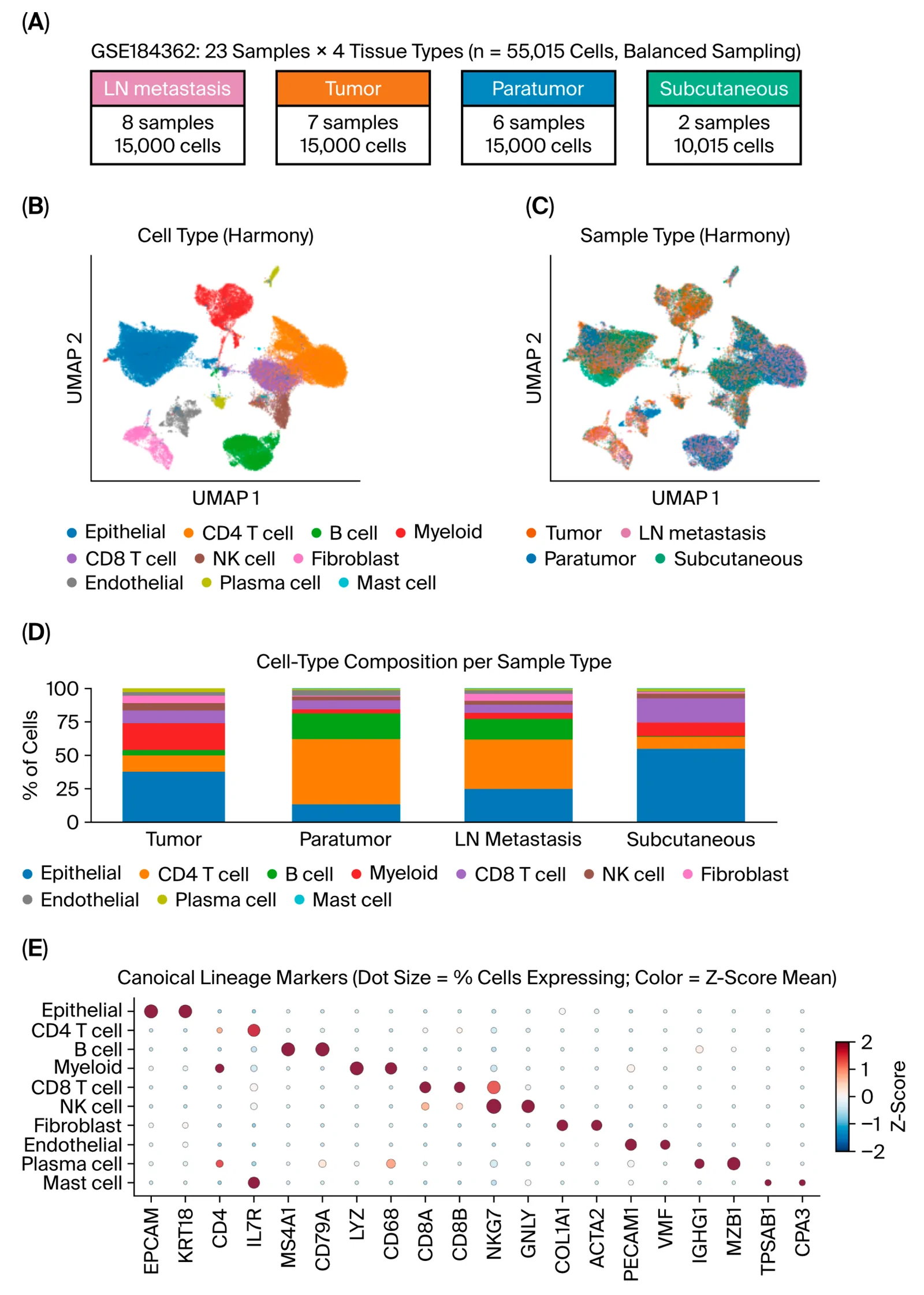
Single-cell atlas of PTC integrated by Harmony. (**A**) Schematic of the 23-sample, 55,015-cell PTC atlas across four tissue contexts. (**B**) UMAP colored by cell-type lineage. (**C**) UMAP colored by sample tissue origin. (**D**) Stacked bar plot showing cell-type composition per sample tissue. (**E**) Dot plot of canonical lineage markers per cell type.

Single-cell RNA-seq data from 23 PTC samples were processed and integrated using Harmony (v0.0.9) after quality control filtering, yielding a total of 55,015 cells in four tissue contexts: primary tumor, paratumor, lymph node metastasis, and subcutaneous metastasis (Figure 1A). Unsupervised clustering followed by canonical marker annotation identified ten main cell lineages (Figure 1B). The Uniform Manifold Approximation and Projection (UMAP) colored by sample tissue origin is shown in Figure 1C, whereas the cell-type composition in the four tissue contexts is presented in Figure 1D. Lineage marker genes showed highly compartment-specific expression patterns in the integrated UMAP (Figure 1E). Quality control metrics and Harmony integration performance are shown in Supplementary Figures S1 and S2.

**Figure 2:**
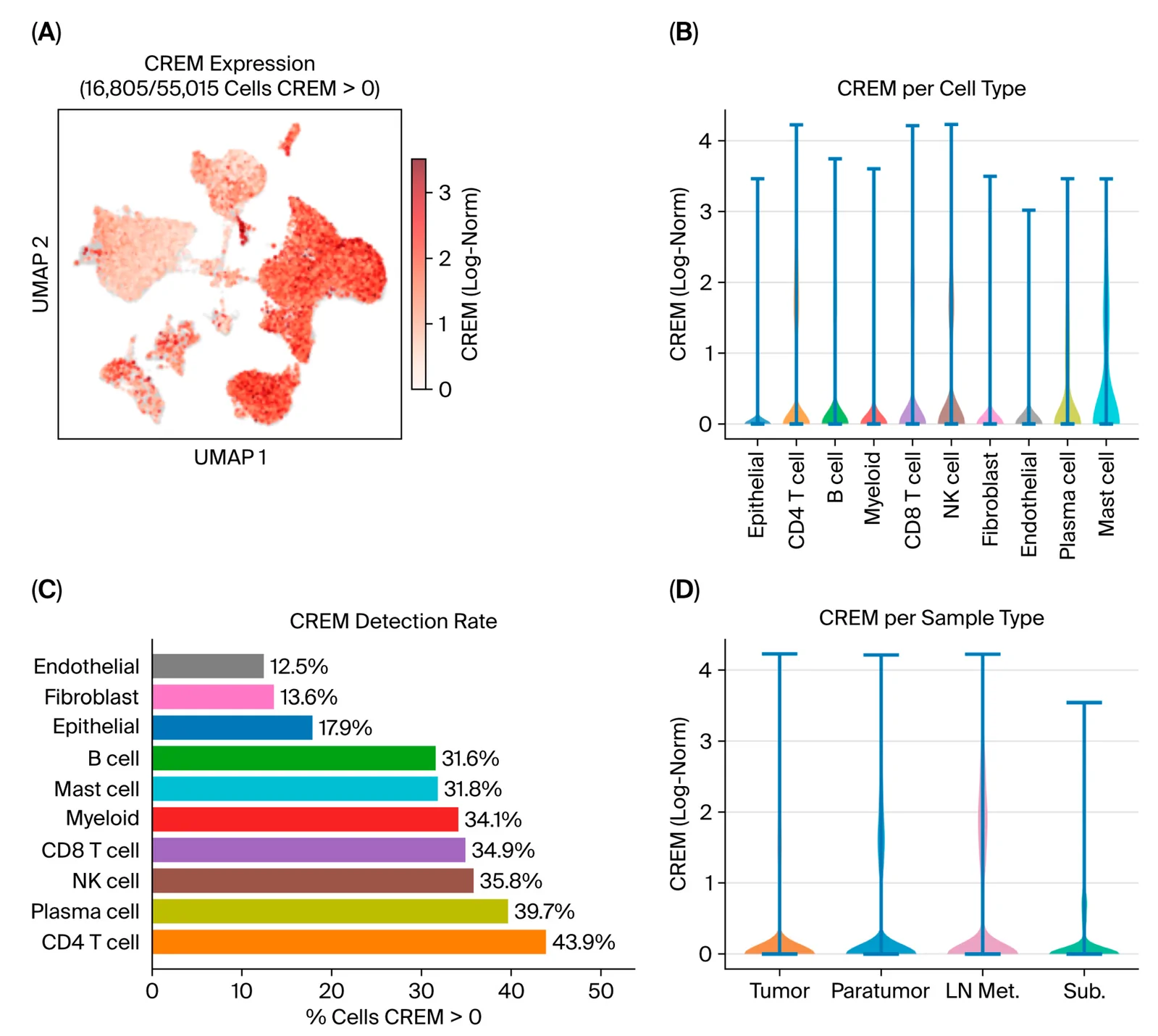
CREM expression across PTC TME lineages. (**A**) Feature plot of CREM expression on the Harmony-integrated UMAP. (**B**) Violin plot of CREM expression per cell type. (**C**) Bar plot of CREM detection rate per cell type. (**D**) Violin plot of CREM expression stratified by sample tissue origin.

CREM expression was observed predominantly in T cells and myeloid cells, with significantly lower expression in epithelial cells (detection rate: CD4 T 43.9%, CD8 T 34.9%, myeloid 34.1% vs. epithelial 17.9%; Wilcoxon *p* < 10^−15^ for each lineage vs. epithelial; Figure 2A–C). CREM levels were similar across all four tissue contexts (Figure 2D). DNA methylation patterns at the CREM locus and their relationship with CREM mRNA expression are shown in Supplementary Figure S3.

### 2.2. CREM Expression Is Strongly Associated with a cAMP_Activated Signature in Tumor-Infiltrating T Cells

**Figure 3:**
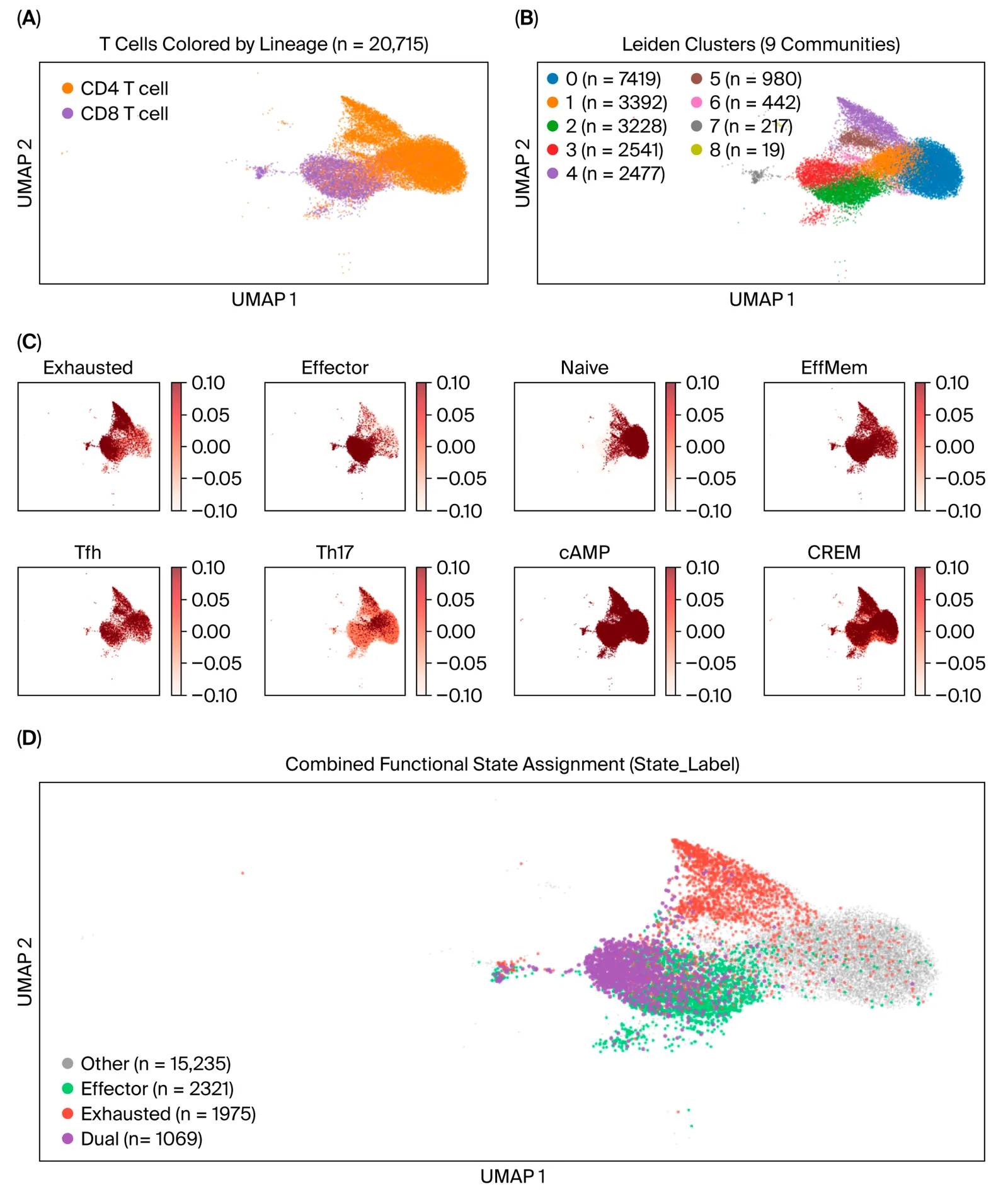
T-cell sub-atlas and functional state classification. (**A**) UMAP of T cells colored by lineage (CD4^+^ and CD8^+^). (**B**) UMAP of T cells colored by Leiden clusters (nine communities). (**C**) Feature plot grid showing multiple T-cell functional state scores. (**D**) UMAP showing classification of T cells into four mutually exclusive functional categories (Exhausted-only, Effector-only, Dual, and Other).

**Figure 4:**
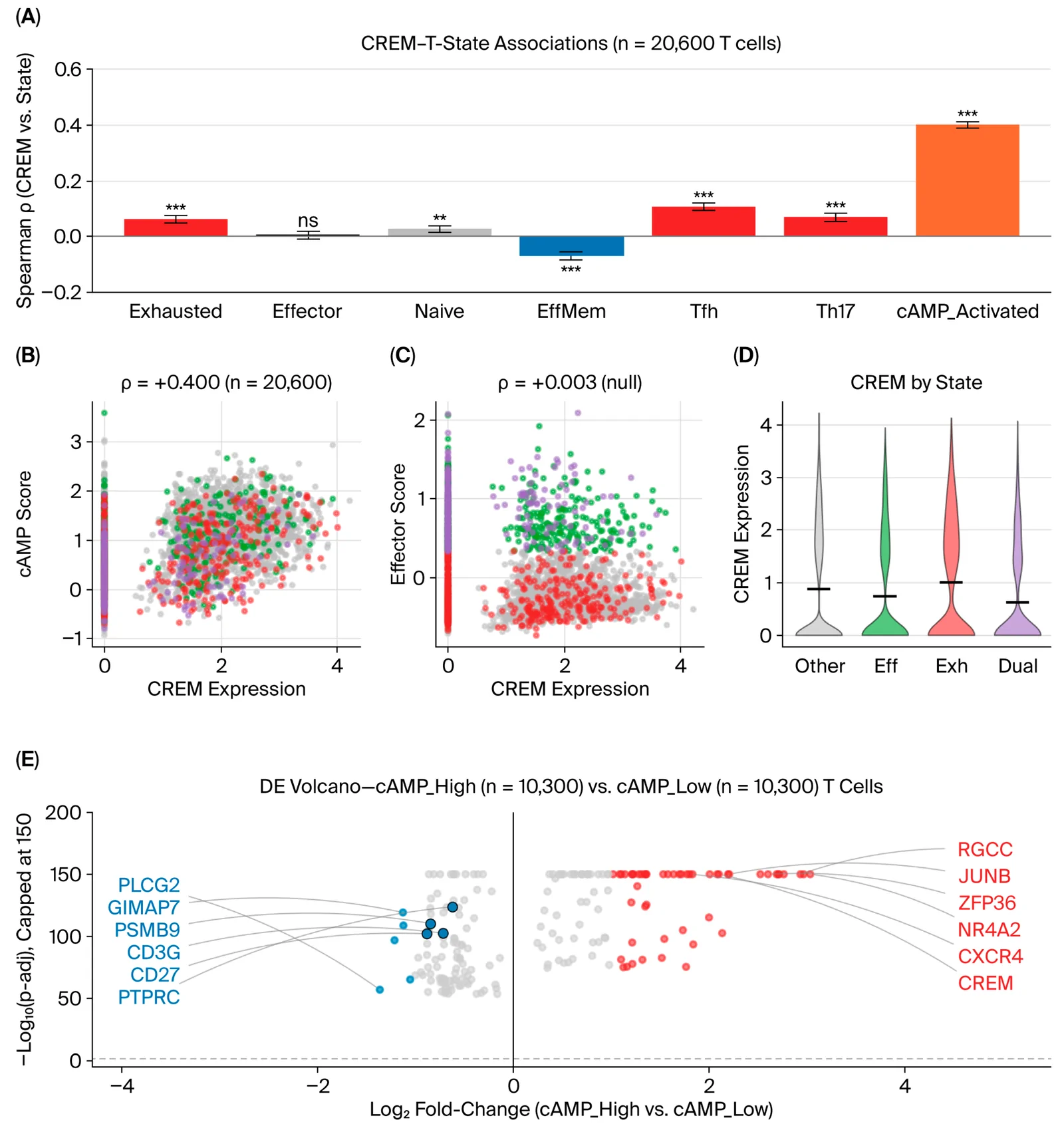
Association of CREM with the cAMP_Activated signature in T cells. (**A**) Bar plot of Spearman correlations (ρ) between CREM and seven T-cell-state scores (n = 20,600). Red bars = significant positive, blue bars = significant negative, gray bars = non-significant or weaker positive correlation (e.g., Naïve, ** *p* < 0.01), the orange bar highlights the cAMP_Activated signature (the focal TCR-driven activation state, ρ = +0.400, *** *p* < 0.001). Asterisks: ns, not significant, ** *p* < 0.01, *** *p* < 0.001. (**B**) Scatter plot of CREM vs. cAMP_Activated score (ρ = +0.400). Points colored by mutually exclusive states: gray (Other), green (Effector-only), red (Exhausted-only), purple (Dual). CREM-high cells show elevated cAMP scores across all states. (**C**) Scatter plot of CREM vs. Effector score (ρ = +0.003, null; negative control). Same color scheme as (**B**). CREM-high cells are not enriched in Effector-only (green), indicating CREM is not merely an Effector marker (key evidence for Section 2.2). (**D**) Violin plots of CREM expression across the four states (colors as in **B**,**C**). (**E**) Volcano plot of differentially expressed genes between cAMP_Activated-high vs. -low T cells (n = 10,300 each). Blue = downregulated, red = upregulated (many with −log_10_ adj. *p* capped at 150), gray = non-significant. Labeled genes are top DE genes.

Focusing on the T-cell compartment, 20,715 T cells were extracted and reclustered. Cells were first separated by CD4/CD8 lineage (Figure 3A) and then divided into nine Leiden communities (Figure 3B). T cells were scored for multiple functional states, visualized as feature plots in Figure 3C. Among all evaluated signatures, CREM showed the strongest positive correlation with the cAMP_Activated signature (Spearman ρ = +0.400, n = 20,600 T cells). This association was stronger than those with other T-cell states (Figure 4A), a pattern further illustrated by the scatter distribution of CREM and cAMP_Activated scores across the full T-cell population (Figure 4B).

In contrast, CREM showed essentially no correlation with the Effector signature, and this lack of association was consistent between CD4^+^ and CD8^+^ T cells (Figure 4C).

The expression of CREM was markedly higher in cAMP_Activated-high cells compared with cAMP_Activated-low cells (Figure 4D,E). Differential expression analysis between these two groups identified six genes that were strongly upregulated in the high group: RGCC, JUNB, ZFP36, NR4A1, NR4A2, and CXCR4 (Figure 4E).

**Figure 5:**
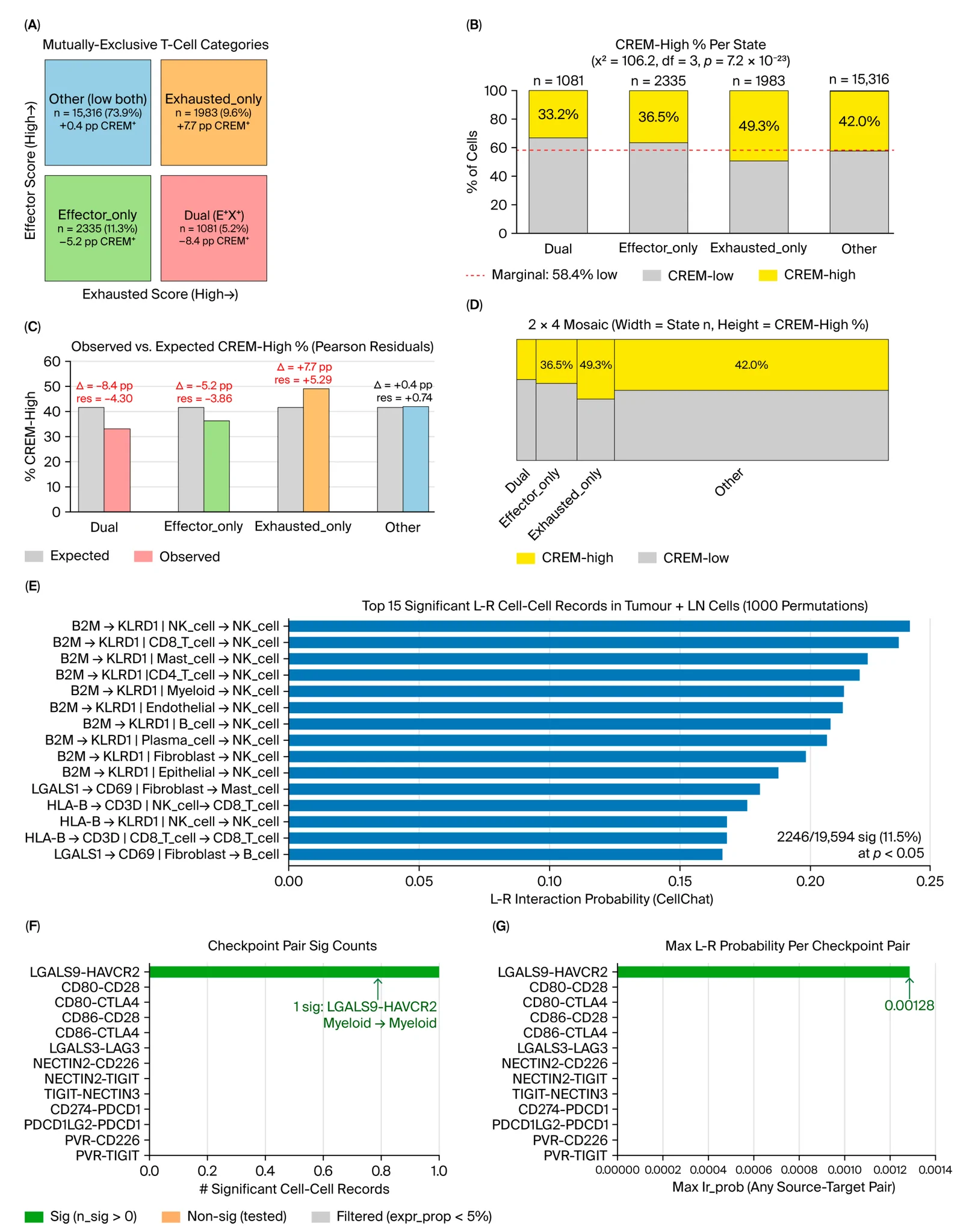
T-cell functional state enrichment and CellChat analysis. (**A**) Schematic of four mutually exclusive T-cell functional categories defined by Effector and Exhausted scores. (**B**) Stacked bar plot showing the proportion of CREM-high cells across functional categories (χ^2^ = 106.2, df = 3, *p* = 7.2 × 10^−23^). (**C**) Bar plot of observed versus expected CREM-high proportions with Pearson’s residuals. Gray bars represent expected percentages under independence; colored bars represent observed percentages (colors match panel (**A**)). (**D**) Mosaic plot of functional category distribution by CREM status. (**E**) Bar plot of the top 15 significant ligand–receptor interactions in tumor + lymph node metastasis samples (1000 permutations). (**F**) Bar plot showing the number of significant interactions across the nine candidate checkpoint pairs that passed the expression-fraction filter (of 13 injected checkpoint pairs). (**G**) Bar plot of maximum ligand–receptor interaction probability for each checkpoint pair. Note: In panels (**F**,**G**), non-significant tested pairs (orange) show no visible bars because their counts and interaction probabilities are zero; filtered pairs (gray) are absent because they fell below the 5% expression-fraction floor and were not tested.

The distribution of T cells across four mutually exclusive functional categories (Exhausted-only, Effector-only, Dual, and Other) is shown in Figure 3D. The T-cell state and the distribution of CREM between functional states are further detailed in Supplementary Figure S4. The coexpression structure of CREM-high T-cell cells was significantly enriched in the Exhausted-only category and depleted in the Effector-only and Dual categories (Figure 5A–D). A detailed single-cell quantification of CREM enrichment within FOXP3^+^ regulatory T cells, including lineage-stratified and stringent dual-positive gating analyses, is provided in Supplementary Figure S5.

### 2.3. The cAMP_Activated Signature Reflects NFκB-Associated Immediate Early Activation Rather than Canonical CAMP Signaling

Although the signature was initially termed cAMP_Activated, we evaluated its biological interpretation by scoring T cells with externally published sets of cAMP-associated genes. These external signatures showed minimal correlation with CREM expression (Supplementary Table S3). In contrast, CREM expression was positively correlated with the Hallmark TNFα–NFκB signature, and this association persisted after accounting for gene overlap (Supplementary Figure S6). These results suggest that the cAMP_Activated signature primarily captures NFκB-driven immediate early transcriptional responses in T cells.

**Figure 6:**
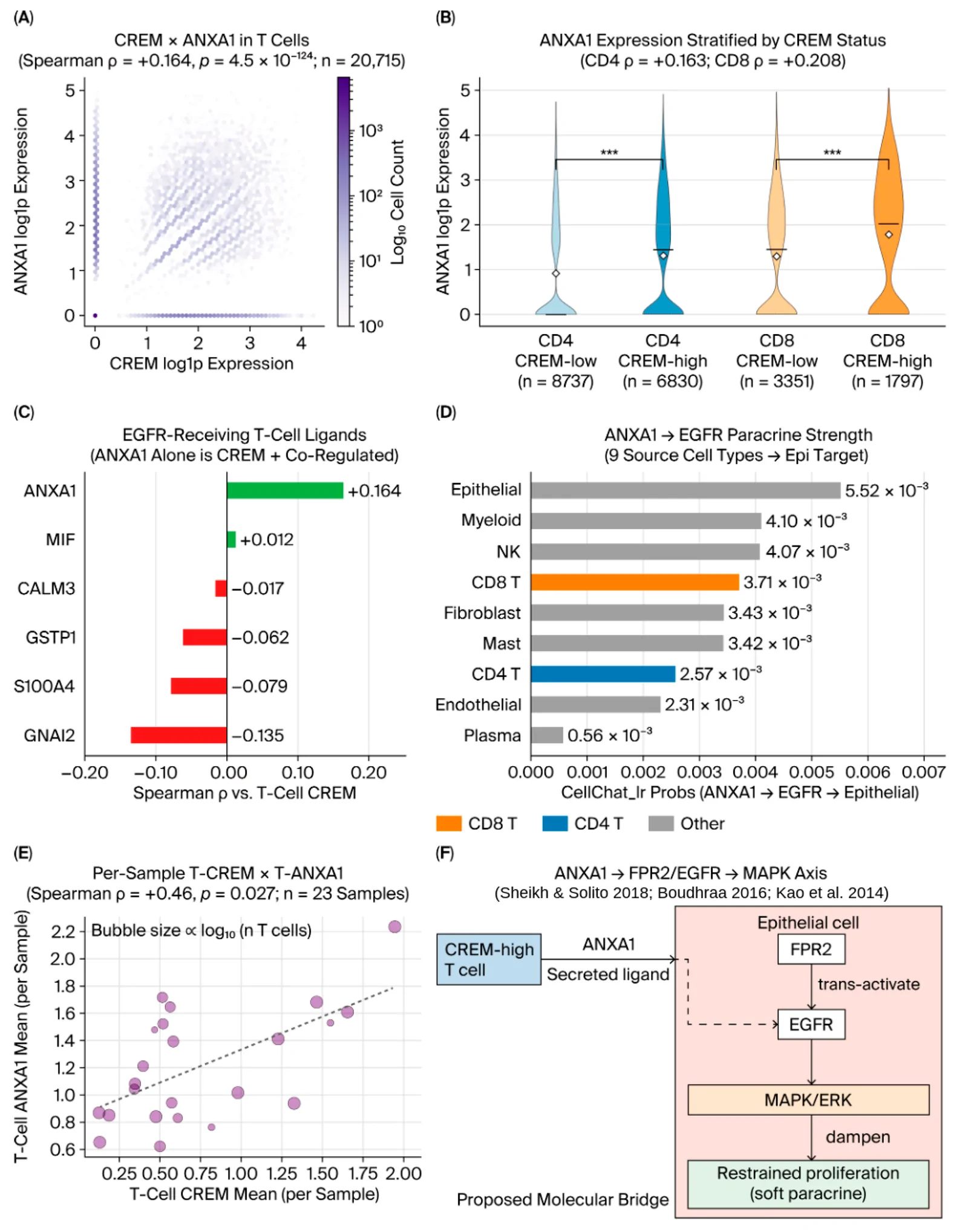
ANXA1–EGFR paracrine axis from CREM-high T cells to epithelial cells. (**A**) Hexbin plot of CREM versus ANXA1 expression in T cells (Spearman ρ = +0.164, *p* = 4.5 × 10^−124^; n = 20,715). Points colored by local density. (**B**) Violin plot of ANXA1 expression in CD4^+^ (blue tones) and CD8^+^ (orange tones) T cells stratified by CREM status. (**C**) Bar plot of Spearman’s correlations between CREM and six EGFR-targeting T-cell ligands. Green bars indicate positive correlations; red bars indicate negative correlations. (**D**) Bar plot of CellChat interaction probabilities for ANXA1–EGFR signaling from different source cell types (orange: CD8 T; blue: CD4 T; gray: Other). (**E**) Scatter plot of mean T-cell CREM versus mean T-cell ANXA1 across 23 samples (bubble size proportional to log_10_ number of T cells). (**F**) Schematic of the proposed ANXA1–EGFR/FPR2 paracrine interaction [14,15,16]. ***: *p* < 0.001 (Extremely statistically significant).

Because the six-gene IEG signature was derived from the same cohort in which the CREM association was estimated, the internal correlation (ρ = +0.400) is an in-sample estimate. We therefore validated the association in an independent cohort, GSE193581 (seven PTC tumors). After library-size normalization (CPM, log1p), the CREM–IEG correlation was replicated at the single-cell level (n = 5454 T cells, ρ = 0.319, *p* = 7.7 × 10^−129^) and at the patient level (n = 7, ρ = 0.857, *p* = 0.014). Leave-one-patient-out cross-validation within the discovery cohort yielded a stable correlation range (ρ = 0.371–0.422, median 0.408). For the per-sample T-CREM × T-ANXA1 correlation (Figure 6E), the patient-level estimate (collapsing each patient’s tissue samples, n = 11) remained positive (ρ = +0.58, *p* = 0.060), consistent with the direction of the sample-level estimate (n = 23, ρ = +0.46, *p* = 0.027) but not reaching significance at the smaller independent n. Leave-one-sample-out analysis showed that the direction was stable to every single-sample deletion (ρ range 0.382–0.523; *p* range 0.012–0.079). We report the per-patient trend in Figure 6E as hypothesis-generating. To further test whether the CREM-associated transcriptional program is cohort-specific, we performed a genome-wide CREM-detected vs. undetected differential expression analysis in GSE193581, followed by gene-set enrichment testing. Because the CREM median in log1p(CPM) is 0, the contrast is CREM-detected vs. undetected rather than high vs. low expression. At the cell level, all six IEG-signature genes were upregulated in CREM-detected cells (FDR < 0.05), and the six-gene set was strongly enriched among upregulated genes (permutation Z = 7.45, *p* = 0.0011). Critically, this enrichment was confirmed at the patient level (n = 7) using two approaches: (a) pseudobulk permutation testing (six-gene IEG Z = 9.87, NR4A Z = 10.00, broad-IEG Z = 17.94, TCR-activation Z = 7.99; all *p* < 0.0001), and (b) a per-patient effect-size Wilcoxon test (six-gene IEG median log2FC = 1.22, *p* = 0.016; NR4A median = 0.86, *p* = 0.031; broad-IEG median = 0.52, *p* = 0.016; TCR-activation median = 0.50, *p* = 0.016). We explicitly note that the six-gene IEG panel was originally derived from a CREM-positive vs. negative T-cell contrast, so its enrichment is partly expected replication; the genuinely non-circular support comes from the literature-derived broad-IEG (27 genes) and TCR-activation (14 genes) sets, which were not derived from our data and still show strong enrichment. These results establish that CREM-detected T cells in an independent cohort are enriched for a TCR-driven immediate early transcriptional program, supporting the reinterpretation of CREM as a marker of TCR-driven IEG activation rather than a cAMP-specific signal.

### 2.4. Candidate NECTIN/PVR Checkpoint Interactions Do Not Reach Significance in Permutation-Controlled CellChat Analysis

Cell–cell communication analysis was performed using CellChat on tumor and lymph node metastasis samples with 1000 permutations. The analysis identified multiple high-confidence ligand–receptor interactions, including B2M–KLRD1 and HLA-B–CD3D pairs (Figure 5E). However, when focusing on the nine candidate immune checkpoint interactions that passed the 5% expression-fraction floor (of 13 checkpoint ligand–receptor pairs injected into the resource to guarantee testing; the remaining four—PVR–TIGIT, PVR–CD226, CD274–PDCD1, and PDCD1LG2–PDCD1—were filtered because PVR/CD274/PDCD1LG2 fell below the expression threshold), pairs involving NECTIN2 or PVR with TIGIT or CD226 did not reach statistical significance (Figure 5F). These pairs also exhibited very low maximum interaction probabilities (Figure 5G). The only checkpoint interaction that was significant was LGALS9–HAVCR2, which was limited to myeloid-to-myeloid signaling (Figure 5F,G).

To test whether CREM-high T cells preferentially signal to epithelial EGFR, we performed differential communication analysis comparing CREM-high versus CREM-low T cells as senders. The ANXA1–EGFR interaction strength did not differ significantly between CREM-high and CREM-low T cells (paired Wilcoxon across five evaluable samples, *p* = 0.63; mean lr_means 0.845 vs. 0.816, Supplementary Table S14). With only five evaluable paired samples, this test is underpowered, so we report the result as “no significant difference detected” rather than evidence of absence. Across all source compartments, the ANXA1–EGFR pair reached permutation significance for CD8 T cells (*p* < 0.01), NK cells (*p* < 0.01), myeloid cells (*p* < 0.01), fibroblasts (*p* = 0.01, FDR = 0.02), and epithelial autocrine signaling (*p* < 0.01), but not for CD4 T cells (*p* = 1.0) (Supplementary Table S12). Thus, the T-cell ANXA1→EGFR signal is driven predominantly by the CD8 compartment, and CREM status does not further stratify communication strength.

#### CREM-High T Cells Upregulate ANXA1 and May Signal to Epithelial Cells via EGFR

We screened for potential interactions between T cells and epithelial cells by examining T-cell-derived ligands that target EGFR. Among the six candidate ligands, ANXA1 showed the strongest positive correlation with CREM in T cells (Figure 6A,C). CREM-high T cells in both CD4 and CD8 lineages expressed higher levels of ANXA1 compared with CREM-low cells (Figure 6B). CellChat analysis supported that CD8^+^ and CD4^+^ T cells can serve as sources of ANXA1 signaling to epithelial EGFR (Figure 6D). In all 23 samples, the mean CREM expression of T cells was positively correlated with the mean expression of ANXA1 of T cells (Figure 6E).

### 2.5. CREM Expression Is Associated with a Prolonged Progression-Free Interval

**Figure 7:**
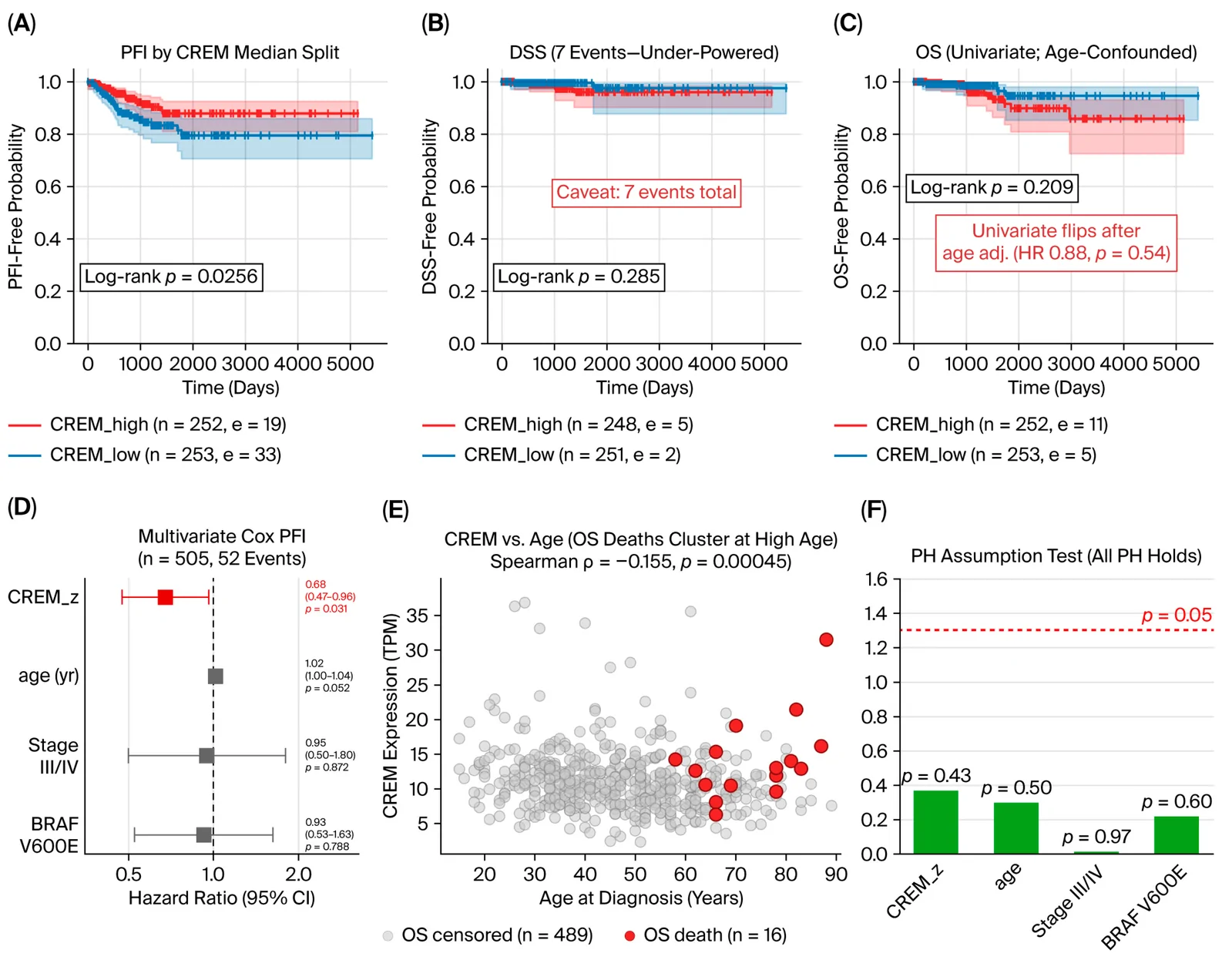
Association of CREM with clinical outcome in TCGA–THCA. (**A**) Kaplan–Meier curve for progression-free interval (PFI) stratified by median CREM expression. (**B**) Kaplan–Meier curve for disease-specific survival (DSS; under-powered, only seven events). (**C**) Kaplan–Meier curve for overall survival (OS). (**D**) Forest plot of multivariate Cox regression for PFI. Vertical dashed line indicates HR = 1. (**E**) Scatter plot of CREM expression versus patient age at diagnosis (gray = OS censored; red = OS death). (**F**) Bar plot of Schoenfeld residual-based proportional hazards (PH) assumption tests. Red horizontal dashed line indicates the *p* = 0.05 significance threshold (all covariates pass the PH assumption).

In the TCGA–THCA cohort, increased expression of CREM was associated with a prolonged progression-free interval (Figure 7A,D). This association was significant in the univariate model and at the border of significance in the multivariate model adjusting for age, stage, and BRAF V600E status (HR 0.56, 95% CI 0.31–1.00, *p* = 0.050; Supplementary Table S8), and remained directionally consistent after further adjustment for sex (HR 0.59, *p* = 0.067), in a stage-free sensitivity model (age, sex, BRAF V600E; HR 0.58, *p* = 0.066), and for the continuous BRAF-RAS score (HR 0.55, *p* = 0.056) (all sensitivity models in Supplementary Table S9). To address the structural collinearity between age and AJCC stage, the protective association was significant in patients <55 years (HR 0.41, 95% CI 0.18–0.92, *p* = 0.031) but not in those ≥55 years (HR 0.83, 95% CI 0.38–1.83, *p* = 0.64); the CREM × age-group interaction was not significant (*p* = 0.22) (age-stratified and interaction models in Supplementary Table S9). The protective association remained directionally consistent across the strata defined by immune infiltration and tumor purity (Table 1, Supplementary Table S5). With 52 PFI events and four covariates, the events-per-variable ratio was 13; disease-specific survival (seven events) was analyzed only exploratorily (Figure 7B). Overall survival differences were largely attributable to age confounding (Figure 7C,E). Schoenfeld residual plots confirmed the proportional hazards assumption for all models (Figure 7F, Supplementary Table S10). CREM expression was negatively correlated with Hallmark E2F and MYC target signatures (Supplementary Tables S4 and S7). Bulk-level immune deconvolution (Supplementary Table S6) and sensitivity analyses are detailed in Supplementary Figures S6–S8.

To test whether the CREM association reflects BRAF-RAS-driven differentiation rather than mutation status per se, we computed the 71-gene BRAF-RAS score (BRS) and 16-gene thyroid differentiation score (TDS) as mean z-scores of log2(TPM+1), restricting the analysis to the 505 primary tumor samples. CREM expression correlated positively with BRS (Spearman ρ = 0.38, *p* = 1.1 × 10^−18^) and more strongly with TDS (ρ = 0.57, *p* = 7.7 × 10^−44^). Adding continuous BRS to the multivariate Cox model did not materially change the CREM association (HR 0.55, 95% CI 0.30–1.01, *p* = 0.056), and BRS itself was not independently associated with PFI (*p* = 0.58). Within BRAF V600E-mutant tumors, CREM remained correlated with BRS (ρ = 0.65, *p* = 3.0 × 10^−35^). Single-cell-level genotyping was not feasible because 3′-tag scRNA-seq does not cover the BRAF/RAS mutation sites; patient-level genotyping of the discovery cohort (seven BRAF V600E, four wild-type, all RAS wild-type) is provided in Supplementary Table S11.

To assess the downstream plausibility of epithelial EGFR engagement, we scored epithelial cells for Hallmark KRAS_SIGNALING_UP and PI3K_AKT_MTOR and correlated patient-level means (collapsing all tissue samples of each patient) with matched T-cell ANXA1 and CREM. At the patient level (n = 10 patients with both epithelial and T cells), KRAS_SIGNALING_UP showed a negative trend against T-cell ANXA1 (ρ = −0.49, *p* = 0.15) that, although not statistically significant at this sample size, is directionally consistent with the hypothesis that T-cell-derived ANXA1 is associated with reduced epithelial MAPK-pathway activity. PI3K_AKT_MTOR showed no correlation with either ANXA1 or CREM (|ρ| ≤ 0.13, *p* ≥ 0.73). We report the patient-level analysis at the true patient *n* (10) rather than the tissue-sample *n* (20) to avoid overstating the number of independent individuals.

### 2.6. Key T-Cell-State Associations Are Robust to Batch Correction

**Figure 8:**
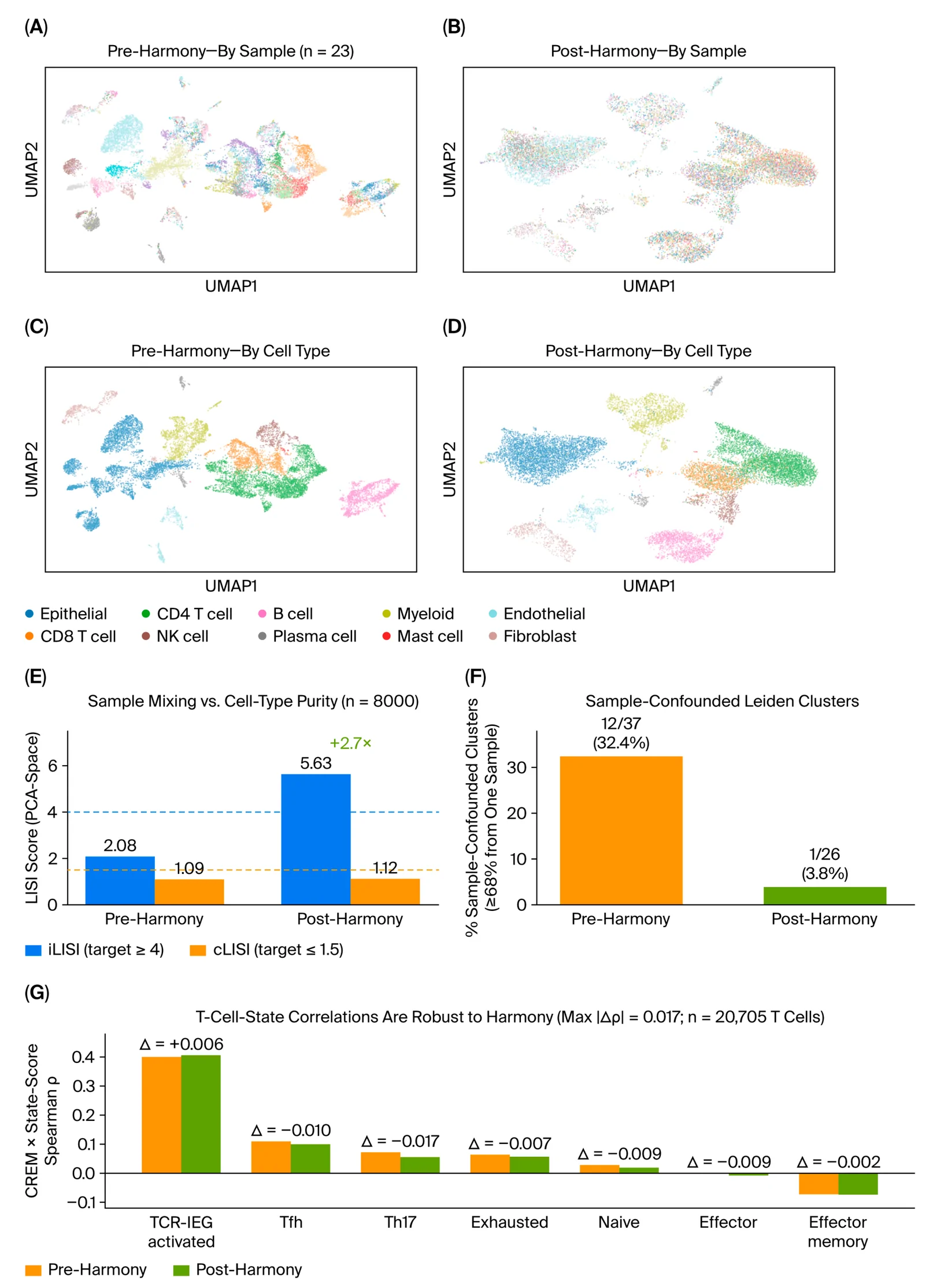
Robustness of integration and T-cell-state findings to Harmony batch correction. (**A**,**B**) UMAP embeddings of all cells before (**A**) and after (**B**) Harmony integration, colored by sample (n = 23 samples). (**C**,**D**) UMAP embeddings before (**C**) and after (**D**) Harmony integration, colored by major cell type. (**E**) Bar plot comparing iLISI (blue) and cLISI (orange) scores before and after Harmony integration (n = 8000 randomly sampled cells). Horizontal dashed lines indicate the predefined target thresholds (blue: iLISI ≥ 4; orange: cLISI ≤ 1.5). Numbers above bars show the observed scores; the +2.7× annotation indicates the improvement in iLISI after integration. (**F**) Bar plot showing the proportion of sample-confounded Leiden clusters (≥68% cells from one sample) before and after integration. (**G**) Bar plot comparing Spearman correlations between CREM expression and seven T-cell functional state scores before and after Harmony correction (n = 20,715 T cells). Δ values indicate the change in correlation after integration (max |Δρ| = 0.017).

Harmony integration improved cross-sample mixing, as evidenced by increased iLISI values and a substantial reduction in sample-confounded Leiden clusters (Figure 8E,F). Per-cell-type analysis confirmed improved mixing for both epithelial and T-cell populations after integration (Supplementary Figure S9). Importantly, the correlations between the functional state signatures of CREM and T cells remained highly consistent before and after Harmony correction (Figure 8G), indicating that the main biological findings are robust to the integration procedure.

## 3. Discussion

PTC is widely characterized as an immunologically “cold” malignancy with low mutational burden, although the precise phenotypic programming of its tumor-infiltrating T cells and their exact contribution to indolent disease progression remain inadequately resolved. In our previous bulk transcriptomic study [12], we associated higher aggregate CREM expression with favorable clinical outcomes and less advanced disease stages; however, such tissue-level data inevitably obscured cell-type-specific behaviors and functional states within the heterogeneous tumor microenvironment. In the present study, to address these limitations, we systematically deconstructed the cellular origins, functional programming, and clinical relevance of CREM at single-cell resolution. Here, our integrated framework demonstrated that, instead of driving a classic cyclic AMP (cAMP)-mediated suppressive program, CREM involved a TCR-driven IEG-activated state in tumor-infiltrating T cells. Crucially, we identified a consistent and modest enrichment of CREM within the FOXP3+ regulatory T-cell compartment (Treg), an effect that remained robust but slightly attenuated under a strict FOXP3+IKZF2+ gate (OR = 1.58), clarifying that the inverse correlations observed at the bulk level reflect a compositional trade-off of microenvironmental cell types rather than intrinsic transcriptomic repression within Tregs. Furthermore, our data propose a novel permutation-controlled paracrine communication axis in which CREM-high T cells actively modulate the regional epithelial compartment through ANXA1-EGFR signaling, providing a robust mechanistic basis for the prolonged PFI observed in bulk clinical cohorts.

This unexpected divergence from the classical functional model of CREM underscores the intense context dependence of T-cell transcriptional wiring between chronic inflammation and oncology. In systemic autoimmune malignancies, most notably SLE, the canonical CREM-alpha isoform acts as a stable epigenetic and transcriptional repressor that directly silences the IL2 promoter while promoting pathogenic Effector cytokine outputs like IL-17 and IL-21 [9,17,18]. In stark contrast, early T-cell receptor (TCR) involvement in acute stimulation settings precipitates a rapid and coordinated influx of immediate early transcription factors downstream of Rel/NF-kB pathways [19,20,21]. Our findings indicate that tumor-infiltrating T cells in PTC utilize this immediate early activation apparatus, rather than transition to sustained chromatin-dependent repressive phenotypes characteristic of autoimmunity. By demonstrating that CREM expression is tracked alongside transient activation signals rather than by terminal dysfunction, these insights provide a mechanistic explanation for why higher CREM expression translates into a less aggressive, lower-proliferation tumor program at the bulk level, distinguishing it from conventional, exhaustion-associated immune checkpoints in thyroid malignancy.

The identification of CREM as an indicator of a TCR-driven IEG activation program, rather than a classical cyclic AMP (cAMP)-mediated suppressive axis, carries profound implications for our understanding of tumor-infiltrating lymphocytes (TILs) in indolent malignancies. Standard oncological paradigms frequently depict TILs along a rigid, linear differentiation trajectory that terminates in irreversible chronic exhaustion [22,23,24]. However, our single-cell deconvolution reveals that CREM expression is robustly correlated with a distinct TCR-driven IEG activation program. Strikingly, although this core transcriptional profile is operationally characterized by the coexpression of RGCC, JUNB, CXCR4, NR4A1, NR4A2, and ZFP36, we underscore the inherent post hoc caveat that this signature expansion was derived from the current cohort, which may partially overestimate correlation magnitudes. Nevertheless, it represents a fundamentally generalized, lineage-independent physiological response that is conserved between both CD4^+^ and CD8^+^ subpopulations within the thyroid tumor microenvironment. Crucially, this coordinated transcriptional response reflects the transient, immediate early downstream effects of antigen receptor engagement rather than a terminal dysfunctional state, an observation that aligns with current research consensus documenting that IEG patterns are highly correlated with acute TCR and antigen receptor activation [25,26,27]. The prevalence of this IEG signature within the PTC microenvironment suggests that, despite its clinically “cold” classification, the tumor milieu does not induce immediate sterile inertia. Instead, T cells within PTC appear to undergo recurrent, localized, and functional antigen receptor engagement, maintaining an early activation program that precedes or bypasses standard exhaustion pathways [28,29].

Importantly, this correlation between CREM and the TCR-IEG-Activated panel represents a lineage-independent phenomenon within the lymphocytic compartment. Our stratified analysis in Section 2.2 demonstrates that the mid-strength correlation between CREM and the activation panel remains highly consistent across both CD4^+^ (rho = +0.390) and CD8^+^ (rho = +0.400) T-cell lineages, with a statistical evaluation confirming no significant difference between the two coefficients (Fisher’s z = −0.68, *p* = 0.495). This lineage-independent conservation (Supplementary Figure S7) raises a critical methodological caution regarding prior tissue-level transcriptomic interpretations. In our previous bulk-level research [12], as well as parallel public datasets, higher global CREM expression was consistently associated with reduced regulatory T-cell (Treg) abundance, leading to the logical post hoc hypothesis that CREM acts as a cell-intrinsic repressor of the Treg lineage. By contrast, our single-cell validation demonstrates that CREM is actually profoundly enriched and exhibits a significantly higher detection rate within strict FOXP3+IKZF2+ Tregs compared with conventional T lineages. Similar confounding phenomena have recently been exposed in other single-cell solid tumor atlases, where apparent gene-signature correlations are inverted upon high-resolution cellular deconvolution [30,31,32]. Consequently, the bulk-level inverse correlation captured in PTC does not signify a cell-intrinsic transcriptional suppression of Tregs by CREM. Rather, it represents a mathematical consequence of microenvironmental cell-type shifts: samples with strong antitumor conventional T-cell activation possess a disproportionately large conventional T-cell pool, which effectively dilutes the relative compositional percentage of Tregs.

Furthermore, our cell–cell communication analysis suggests a candidate intercellular circuit in which tumor-infiltrating T cells—predominantly the CD8 compartment—are a correlated source of ANXA1 signaling toward epithelial EGFR. We note that this T-cell-derived, potentially antiproliferative role contrasts with the predominantly oncogenic, tumor-cell-intrinsic role reported for ANXA1 in other contexts [33,34]. Within the indolent clinical landscape of PTC, such an association is non-canonical and should be regarded as a hypothesis. While this correlative axis may contribute to the favorable prognosis associated with high CREM expression, it does not establish causality. More fundamentally, our genome-wide differential expression and gene-set enrichment analyses in an independent cohort (GSE193581) establish that CREM-detected T cells are enriched for a TCR-driven immediate early transcriptional program (six-gene IEG, NR4A family, broad-IEG, and TCR-activation sets all significantly enriched at the patient level, n = 7). This supports a conceptual reinterpretation of CREM: rather than functioning solely as a cAMP-responsive transcription factor, CREM expression in tumor-infiltrating T cells marks recent TCR-driven IEG activation. Under this reinterpretation, the ANXA1–EGFR communication axis is one candidate downstream consequence of that activation state, not the primary claim of the study.

Several limitations warrant recognition. First, the six-gene IEG signature was derived from the same discovery cohort in which the CREM association was estimated, introducing potential circularity. Although we mitigated this by independent validation in GSE193581 and leave-one-patient-out cross-validation, the internal effect size (ρ = +0.400) should be interpreted as an in-sample estimate. We further note that the genome-wide CREM-detected vs. undetected enrichment of the six-gene IEG panel in GSE193581 is partly expected replication (the panel was derived from a CREM-positive vs. negative contrast); the genuinely non-circular support comes from the literature-derived broad-IEG (27 genes) and TCR-activation (14 genes) sets, which were not derived from our data and still show strong patient-level enrichment (n = 7, pseudobulk permutation Z = 17.94 and Z = 7.99 respectively, both *p* < 0.0001). Second, the patient-level inference is limited to 11 individuals (20,715 cells), the per-sample correlation (Figure 6E) is a hypothesis-generating trend at the patient level (n = 11, ρ = +0.58, *p* = 0.060), and the subcutaneous metastasis subgroup comprises only two samples (10,015 cells after balanced down sampling), so subgroup-specific conclusions are exploratory. Third, the multivariate PFI association, while directionally robust across sensitivity models, sits at the border of statistical significance (*p* = 0.05–0.07) given the modest number of progression events (n = 52), and should be confirmed in larger cohorts. Fourth, the proposed functional axes—the TCR-IEG-driven induction of CREM and the paracrine ANXA1–EGFR crosstalk—are derived from transcriptomic inference and computational modeling rather than direct experimental perturbation. Future validation using in vitro co-culture assays and spatial transcriptomics will be essential to establish these interactions causally.

## 4. Materials and Methods

### 4.1. Data Sources for Single-Cell RNA-Sequencing

RNA-seq data were obtained from the GEO accession GSE184362 [13], which comprised 23 samples from 11 patients with PTC in four tissue contexts: primary tumor, paratumor, lymph node metastasis, and subcutaneous metastasis. An independent single-cell RNA-seq validation cohort was retrieved from GEO accession GSE193581 [35], including 10 anaplastic thyroid carcinoma tumors, 7 PTC tumors, and 6 adjacent normal thyroid tissues. Only the 7 PTC tumor samples from this dataset were used in the present study for external validation of the CREM-associated T-cell transcriptional program. Bulk transcriptomic, clinical, and DNA methylation data for TCGA–THCA were retrieved from Genomic Data Commons, with PFI endpoints curated from the PanCanAtlas TCGA-CDR resource [36]. Gene sets were obtained from MSigDB v2024.1 [37]. Detailed data sources and preprocessing procedures are described in the Supplementary Methods.

### 4.2. Single-Cell Data Processing and Batch Correction

Raw count matrices for single-cell data processing and batch correction were subjected to quality control filtering based on the number of detected genes and the mitochondrial content, followed by normalization, log-transformation, highly variable gene selection, and principal component analysis. Harmony integration [38] was applied using the sample as a batch key to correct for technical variation across samples. Unsupervised Leiden [39] clustering was performed and cell lineages were annotated based on canonical marker gene scoring. Full QC thresholds, integration parameters, and quality assessment metrics are provided in the Supplementary Methods.

Quality control retained cells with ≥200 detected genes, <7500 genes per cell (upper-bound doublet proxy), <20% mitochondrial transcripts, and ≥500 total counts; genes detected in ≥3 cells were kept. Clustering used the Leiden algorithm at resolution 1.0 for both the full atlas and the T-cell sub-atlas. Doublet control was applied at two levels: an upper-bound gene-count filter (<7500 genes) as an initial doublet proxy, and cluster-level resolution during manual lineage annotation, in which clusters showing a balanced coexpression of canonical markers from distinct lineages were resolved by assigning each to its dominant lineage by marker score, while one small cluster (236 cells) without a dominant lineage was removed as doublet-enriched. The final atlas comprised 37 clusters, all 100% lineage-pure. Manual annotation was cross-validated against SingleR (Human Primary Cell Atlas reference), yielding 92.8% concordance (n = 54,045 cells across seven lineages, Supplementary Table S13).

### 4.3. T-Cell Sub-Atlas and Functional State Scoring

CD4^+^ and CD8^+^ T cells were isolated to construct a lineage-specific sub-atlas with recomputed dimensionality reduction. Six functional canonical T-cell states (Exhausted, Effector, Naïve, Effector Memory, Tfh, and Th17) [40,41,42,43,44] were scored using published gene signatures. A post hoc six-gene NFκB-IEG-activated signature was derived from differentially expressed genes associated with CREM within this cohort and externally validated against an independent reference gene set related to cAMP and NFκB. FOXP3^+^ regulatory T cells were identified using a canonical five-gene Treg panel. The details of the gene set and the scoring parameters are provided in Supplementary Table S1 and the Supplementary Methods.

### 4.4. Cell–Cell Communication Analysis

Cell–cell communication was inferred on primary tumor and lymph node metastasis samples using the CellChat [45] model implemented in liana-py (version 1.7.3), with 1000 permutations to control false discovery. A consensus ligand–receptor resource was supplemented with manually curated immune checkpoint interaction pairs. Population-level significant interactions were identified after multiple testing correction. Detailed analysis parameters and custom ligand–receptor pairs are provided in the Supplementary Methods and Supplementary Table S2.

### 4.5. Survival Analysis and Pathway Scoring

Cox proportional hazards models were used to evaluate the association between CREM expression and PFI, adjusted for age, sex, AJCC stage, and BRAF V600E status. Because AJCC 8th-edition staging uses age 55 as a boundary, age and stage are structurally collinear; we therefore additionally fitted age-stratified models (<55 vs. ≥55 y), a CREM × age-group interaction term, a sex-adjusted model, and a stage-free sensitivity model (age, sex, BRAF V600E).

### 4.6. Statistical Analysis and Reproducibility

Spearman’s rank correlation, Fisher’s exact test, the Cochran–Mantel–Haenszel test, and the Wilcoxon rank-sum test with Benjamini–Hochberg correction were applied as appropriate. All analyses were conducted with fixed random seeds to ensure reproducibility. Complete software versions, parameter settings, and analysis scripts are available in the Supplementary Methods and the associated GitHub (version v1.0.0, commit ID: 04cd86e) repository (https://github.com/linghao1992-sketch/crem-ptc (accessed on 9 August 2026)). Analyses performed after initial data inspection (post hoc) were the derivation of the six-gene IEG signature, CREM–IEG correlation, CREM-high/low differential cell–cell communication, and epithelial pathway correlation. A priori hypotheses were the CREM–PFI and CREM–T-cell-state associations.

### 4.7. DNA Methylation and Bulk Immune Deconvolution

DNA methylation correlation at the CREM locus and bulk immune deconvolution were performed using MCP-counter [46] and ESTIMATE [47] algorithms as supporting analyses. The complete protocols are described in the Supplementary Methods.

## 5. Conclusions

In this study, we provide a single-cell-resolution architecture of CREM in PTC, redefining it as a marker of an acute, TCR-dependent immediate early T-cell activation program rather than chronic transcriptomic exhaustion or the suppression of cAMP. By disentangling tissue-level compositional biases, we show that CREM is intrinsically enriched in active regulatory T cells and marks a TCR-driven immediate early transcriptional program, as confirmed by genome-wide differential expression and gene-set enrichment in an independent cohort. CREM expression is also associated with a candidate ANXA1–EGFR paracrine communication axis—driven predominantly by CD8 T cells—toward the tumor epithelium, which may contribute to the favorable prognosis associated with high CREM expression.

## Figures and Tables

**Table 1:** Stratified PFI Cox sensitivity for CREM.

Stratum	n	Events	HR	95% CI	*p*-Value	Interaction *p*
Full cohort	505	52	0.677	0.475–0.964	0.031	—
ImmuneScore high	253	30	0.706	0.440–1.134	0.150	0.725
ImmuneScore low	252	22	0.742	0.475–1.158	0.189	0.725
Purity Q1 (low)	127	19	0.769	0.482–1.228	0.271	—
Purity Q2	126	14	0.697	0.411–1.183	0.181	—
Purity Q3	126	7	0.751	0.367–1.539	0.434	—
Purity Q4 (high)	126	12	0.930	0.610–1.417	0.735	—
Purity low (Q1 + Q2)	253	33	0.630	0.397–1.000	0.050	0.655
Purity high (Q3 + Q4)	252	19	0.800	0.498–1.287	0.358	0.655

## Data Availability

The single-cell RNA-sequencing data of the discovery cohort are publicly available in the Gene Expression Omnibus (GEO) under accession number GSE184362; the independent validation cohort data are available under accession number GSE193581. Bulk transcriptomic profiles, clinical follow-up data, and DNA methylation data for the TCGA–THCA cohort were retrieved from the Genomic Data Commons (GDC) portal, with progression-free interval endpoints curated from the PanCanAtlas TCGA-CDR resource. Reference gene sets were obtained from the Molecular Signatures Database (MSigDB) v2024.1. The full analysis code used in this study is openly accessible at https://github.com/linghao1992-sketch/crem-ptc (accessed on 9 August 2026). Processed intermediate data supporting the findings of this work are available from the corresponding author upon reasonable request.

## Supplementary Materials

The following supporting information can be downloaded at: https://www.mdpi.com/article/10.3390/ijms27167387/s1. Reference [48] is cited in Table S8 in the supplementary materials.

## Abbreviations

AJCC	American Joint Committee on Cancer
ANXA1	Annexin A1
cAMP	Cyclic Adenosine Monophosphate
CREM	cAMP-Responsive Element Modulator
EGFR	Epidermal Growth Factor Receptor
GEO	Gene Expression Omnibus
ICB	Immune Checkpoint Blockade
IEG	Immediate Early Gene
PFI	Progression-Free Interval
PTC	Papillary Thyroid Carcinoma
PVR	Poliovirus Receptor
RNA-seq	RNA Sequencing
scRNA-seq	Single-Cell RNA Sequencing
SLE	Systemic Lupus Erythematosus
TCGA	The Cancer Genome Atlas
TCGA–THCA	The Cancer Genome Atlas Thyroid Carcinoma
TCR	T-Cell Receptor
TIGIT	T-Cell Immunoreceptor with Ig and ITIM Domains
TIL	Tumor-Infiltrating Lymphocyte
TME	Tumor Microenvironment
Treg	Regulatory T Cell
UMAP	Uniform Manifold Approximation and Projection
