# Supplementary material for: CREM Marks TCR-Driven T-Cell Activation and Associates with Favorable Prognosis in Papillary Thyroid Carcinoma: A Single-Cell and Bulk Transcriptomic Study
Source: Int J Mol Sci. 2026 Aug 18;27(16):7387. doi: 10.3390/ijms27167387 (PMC13513026; doi:10.3390/ijms27167387)
Supplement: Supplementary file 1 [file ijms-27-07387-s001.zip › ijms-4463250-supplementary/Supplementary File(s)/Supplementary Materials Legend.pdf]

Supplementary Figures

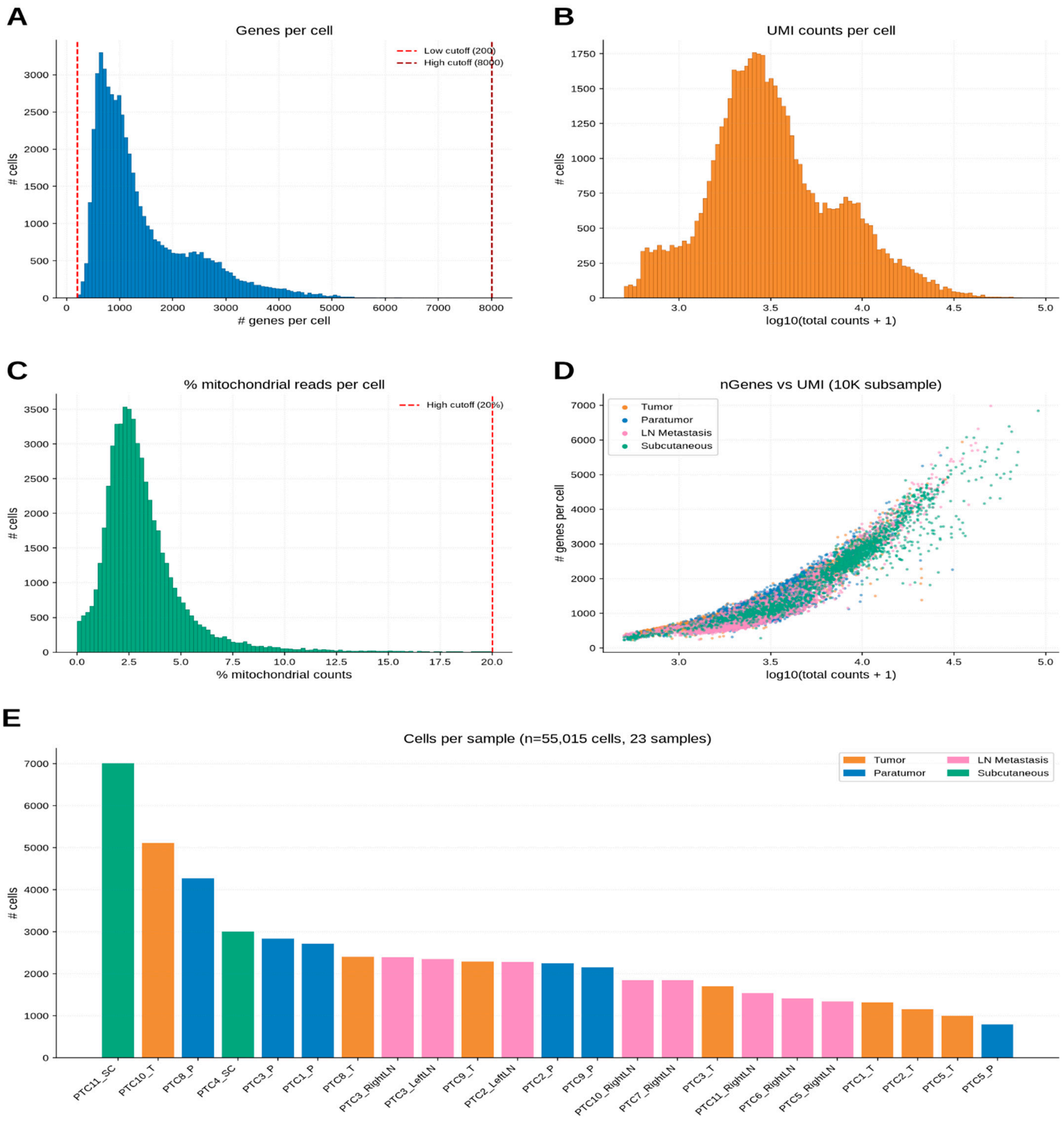

Supplementary Figure S1. Quality control of single-cell RNA-seq data. **(A)** Distribution of genes per cell. Red dashed lines indicate low (200) and high (8000) filtering cutoffs. **(B)** Distribution of $\log_{10}$(UMI counts + 1) per cell. **(C)** Distribution of mitochondrial read percentage per cell. Red dashed line indicates the high cutoff (20%). **(D)** Scatter plot of genes versus UMI counts (10K subsample), colored by sample origin. **(E)** Number of cells per sample after QC (n = 55,015 cells, 23 samples), colored by tissue type (Tumor, Paratumor, LN Metastasis, Subcutaneous).

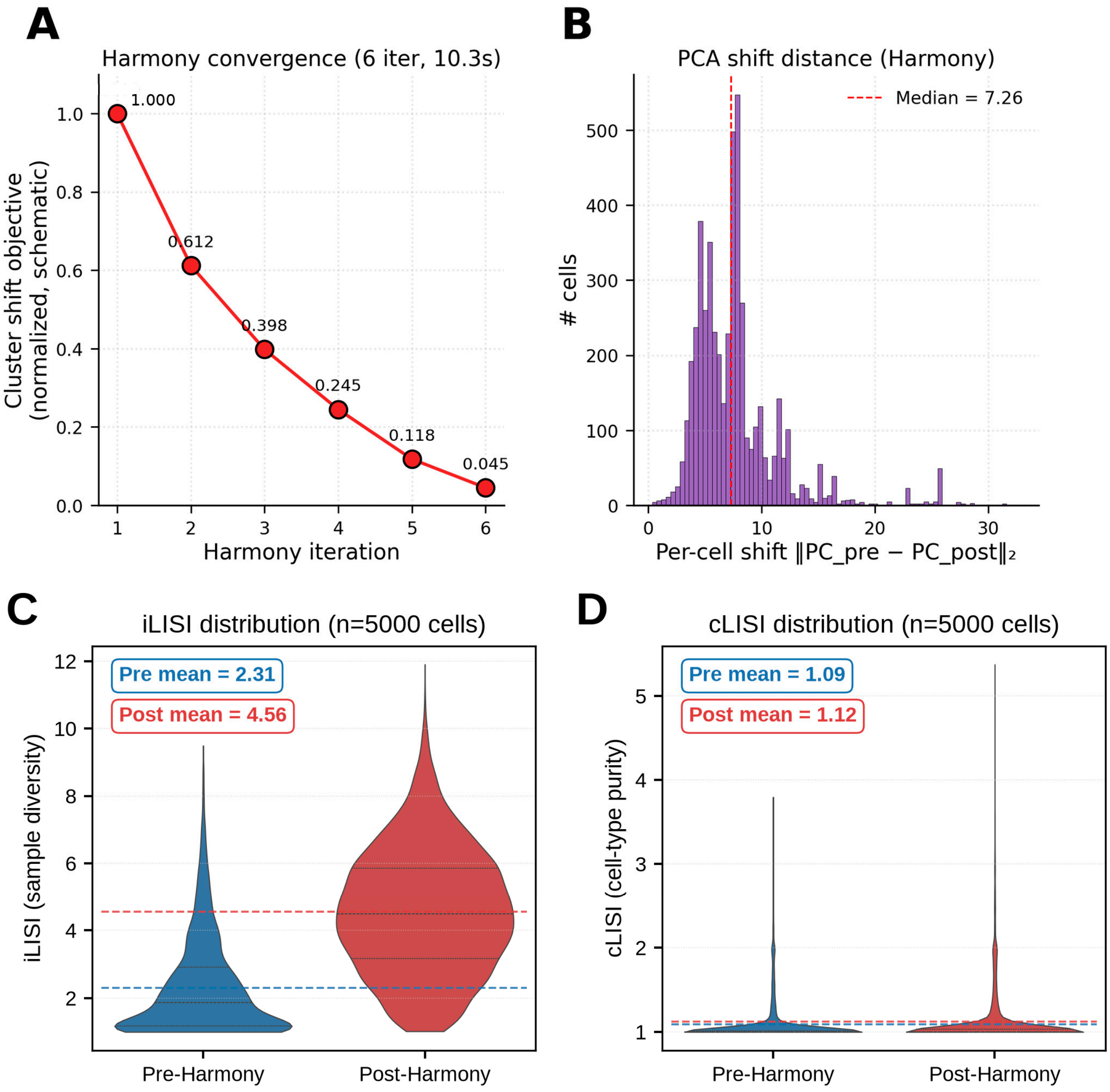

Supplementary Figure S2. Harmony integration diagnostics. (**A**) Harmony convergence trajectory. (**B**) Distribution of per-cell PCA shift distances. (**C**,**D**) Distributions of iLISI and cLISI before and after integration.

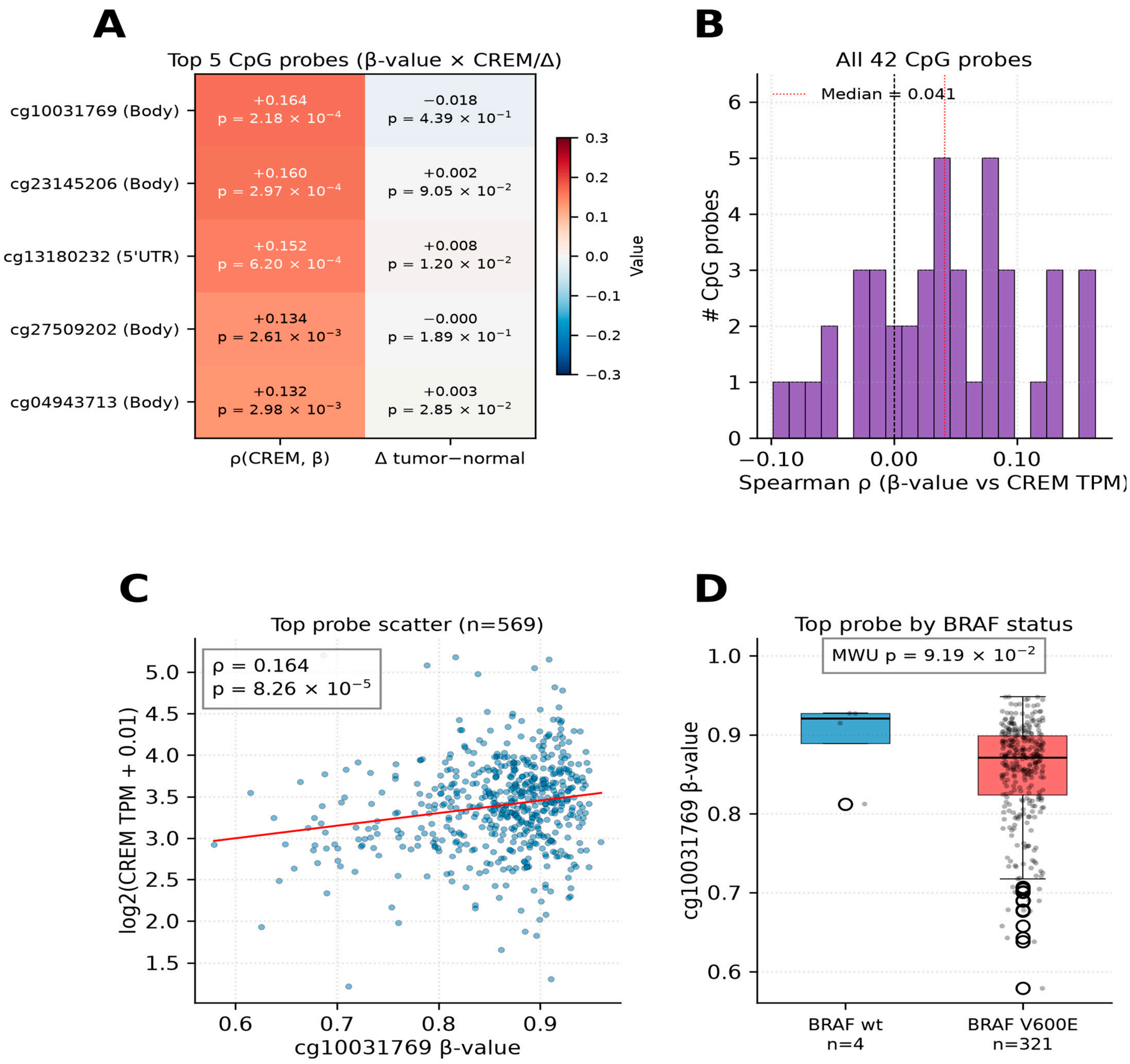

Supplementary Figure S3. DNA methylation at the CREM locus. (**A**) Heatmap of top CpG probes correlated with CREM expression. (**B**) Distribution of Spearman's correlations between all CREM-associated CpG probes and CREM expression. (**C**) Scatter plot of top probe methylation versus CREM expression. (**D**) Box plot of top probe methylation stratified by BRAF V600E status.

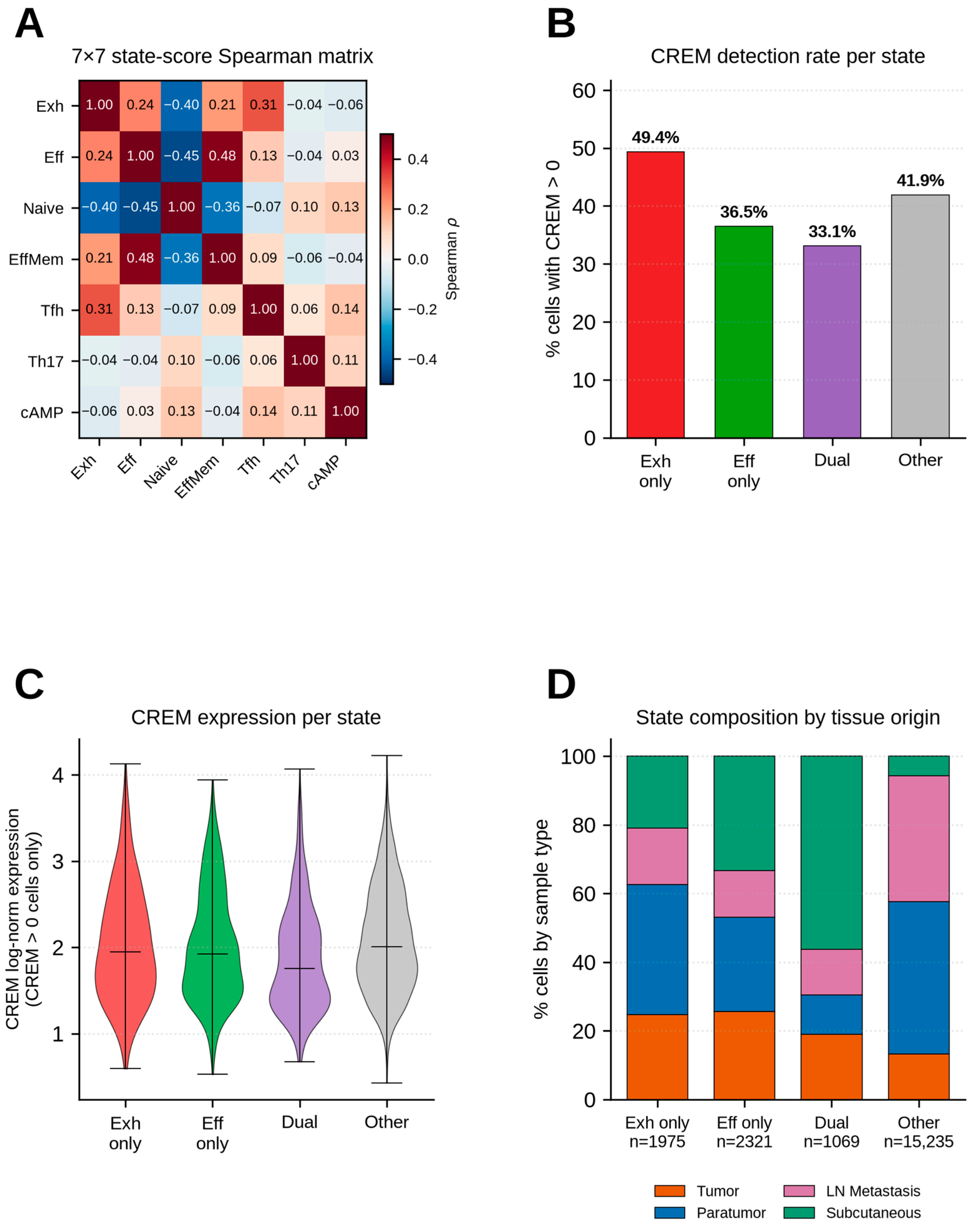

Supplementary Figure S4. T-cell functional state relationships. (**A**) Correlation heatmap of seven T-cell functional state scores. (**B**) Bar plot of CREM detection rate across functional categories. (**C**) Violin plot of CREM expression across functional categories. (**D**) Stacked bar plot of functional category composition by tissue origin.

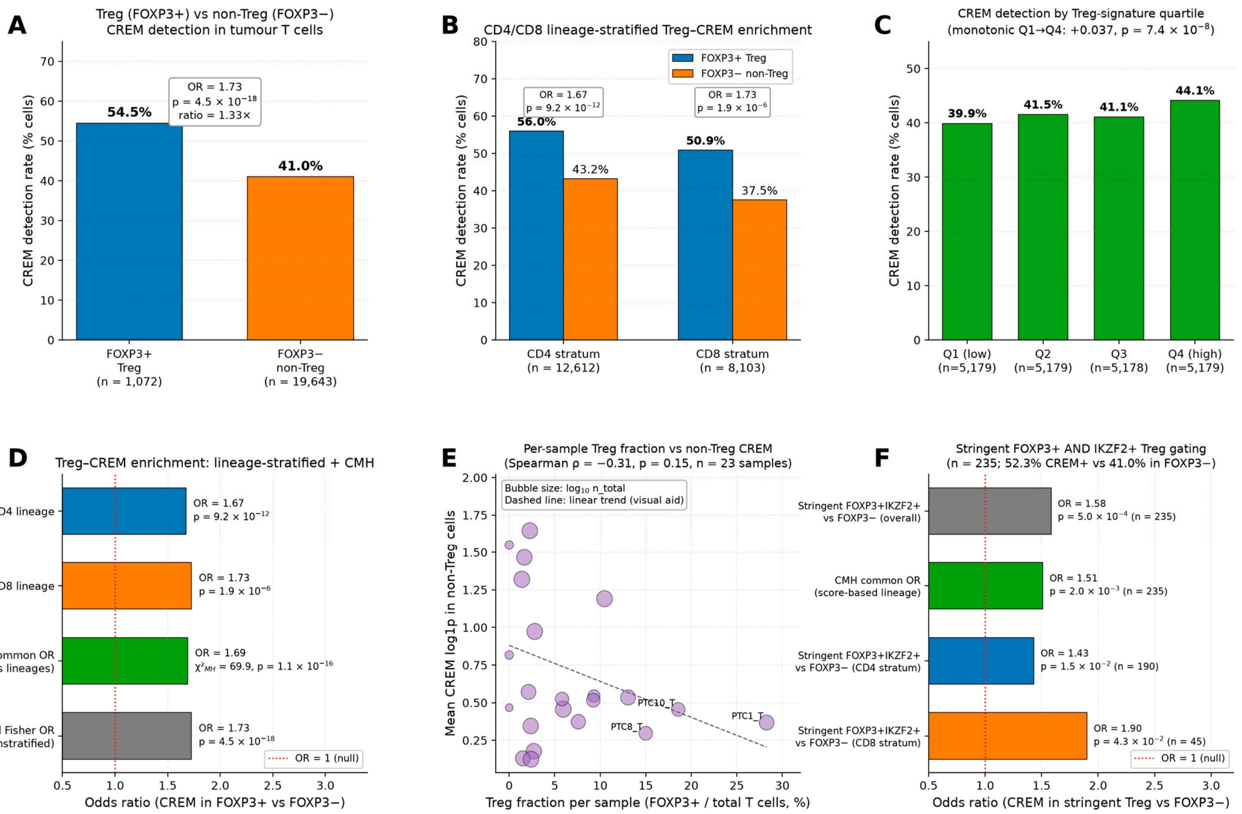

Supplementary Figure S5. CREM enrichment in FOXP3$^+$ regulatory T cells. (**A**–**F**) Bar plots, forest plots, and scatter plots showing CREM detection rates and enrichment odds ratios in FOXP3$^+$ versus FOXP3$^-$ T cells, including lineage-stratified and stringent (FOXP3$^+$IKZF2$^+$) analyses.

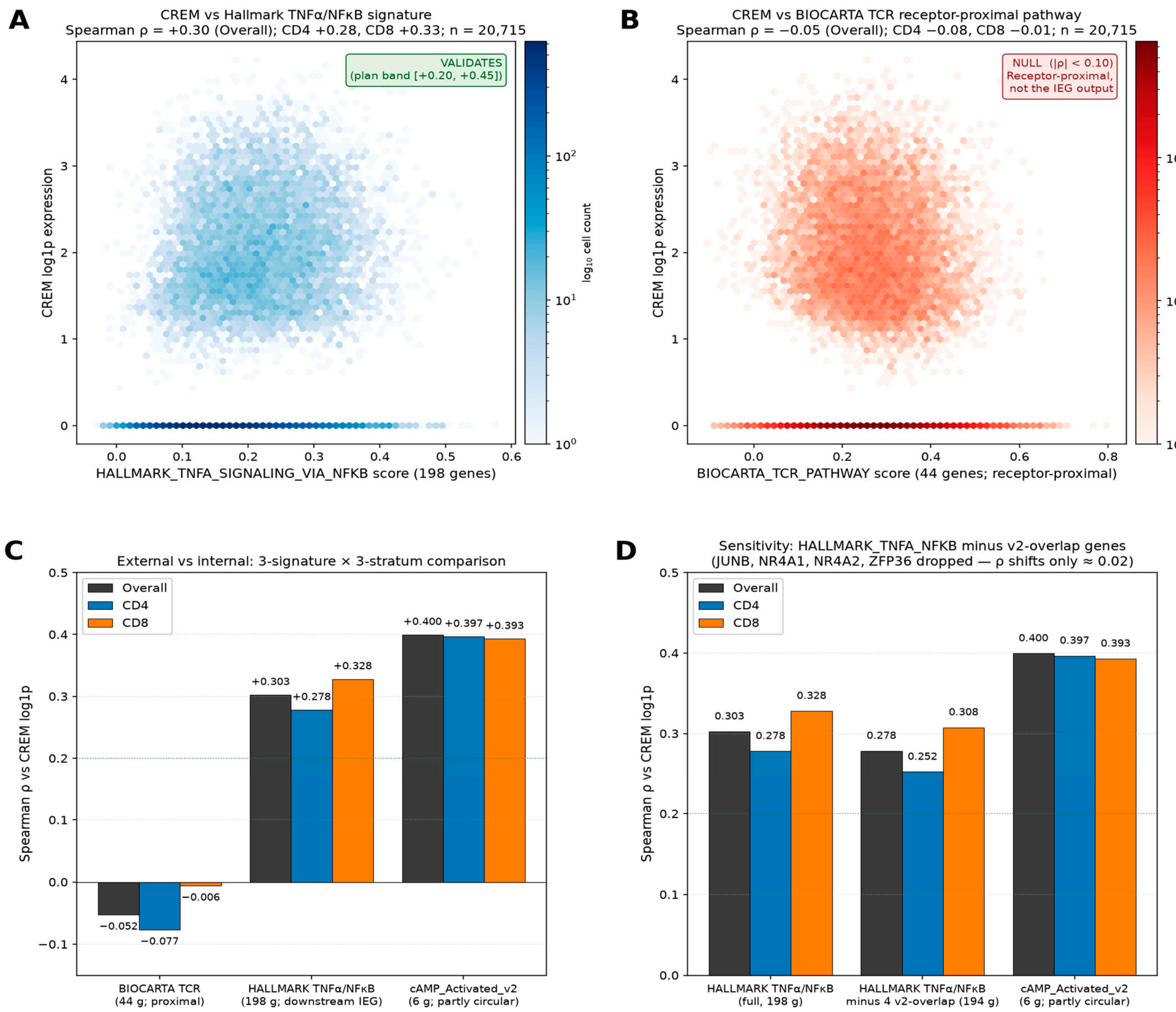

Supplementary Figure S6. External validation of the NFκB-IEG signature. (**A**) Hexbin plot of CREM versus Hallmark TNFα–NFκB score. (**B**) Hexbin plot of CREM versus BIOCARTA TCR signaling score. (**C**) Grouped bar plot comparing correlations across multiple signatures. (**D**) Sensitivity analysis of Hallmark TNFα–NFκB correlation after removing overlapping genes.

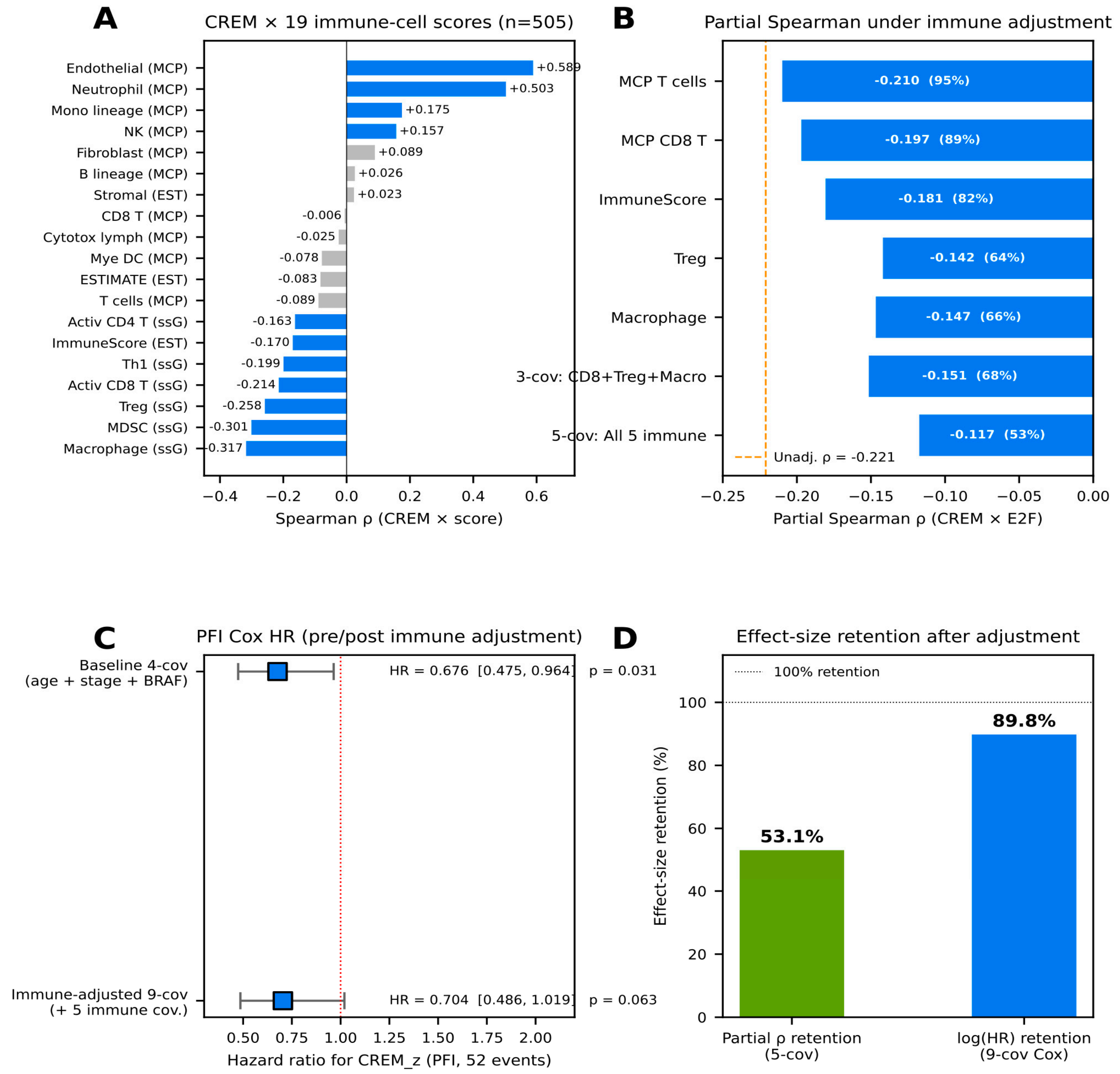

Supplementary Figure S7. Bulk immune deconvolution and sensitivity analyses. (**A**) Heatmap of correlations between CREM and immune cell scores. (**B**) Forest plot of partial correlations between CREM and E2F targets under immune adjustment. (**C**) Comparison of PFI hazard ratios before and after immune adjustment. (**D**) Bar plot of effect size retention after adjustment.

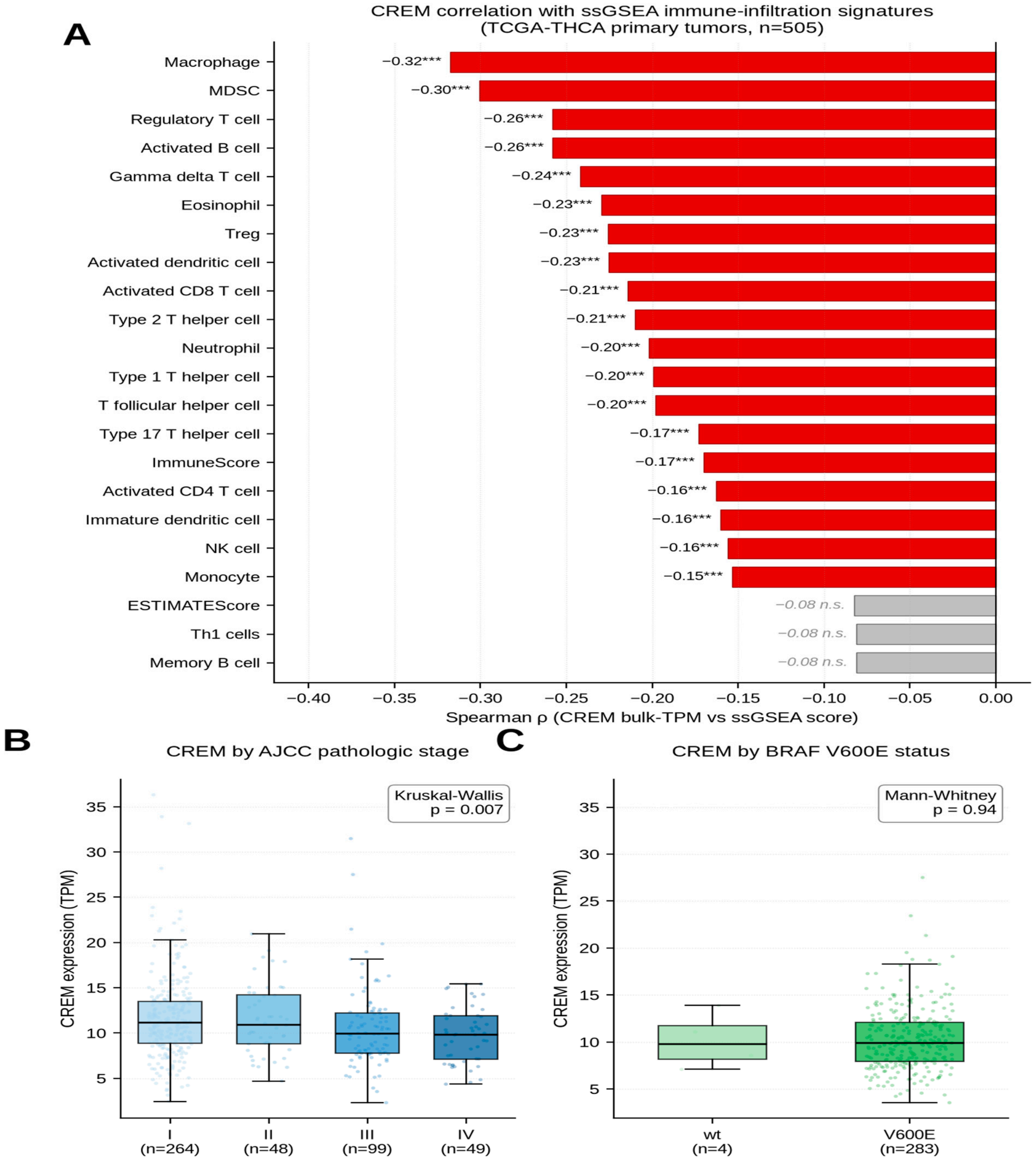

Supplementary Figure S8. Bulk-level immune and clinical correlations. **(A)** Bar plot of Spearman correlations between CREM (bulk-TPM) and ssGSEA immune-infiltration signatures in TCGA-THCA primary tumors (n = 505). Red bars indicate significant negative correlations (***P < 0.001); gray bars indicate non-significant correlations. **(B)** Box plots of CREM expression by AJCC pathologic stage (Kruskal-Wallis p = 0.007). **(C)** Box plots of CREM expression by BRAF V600E status (Mann-Whitney p = 0.94).

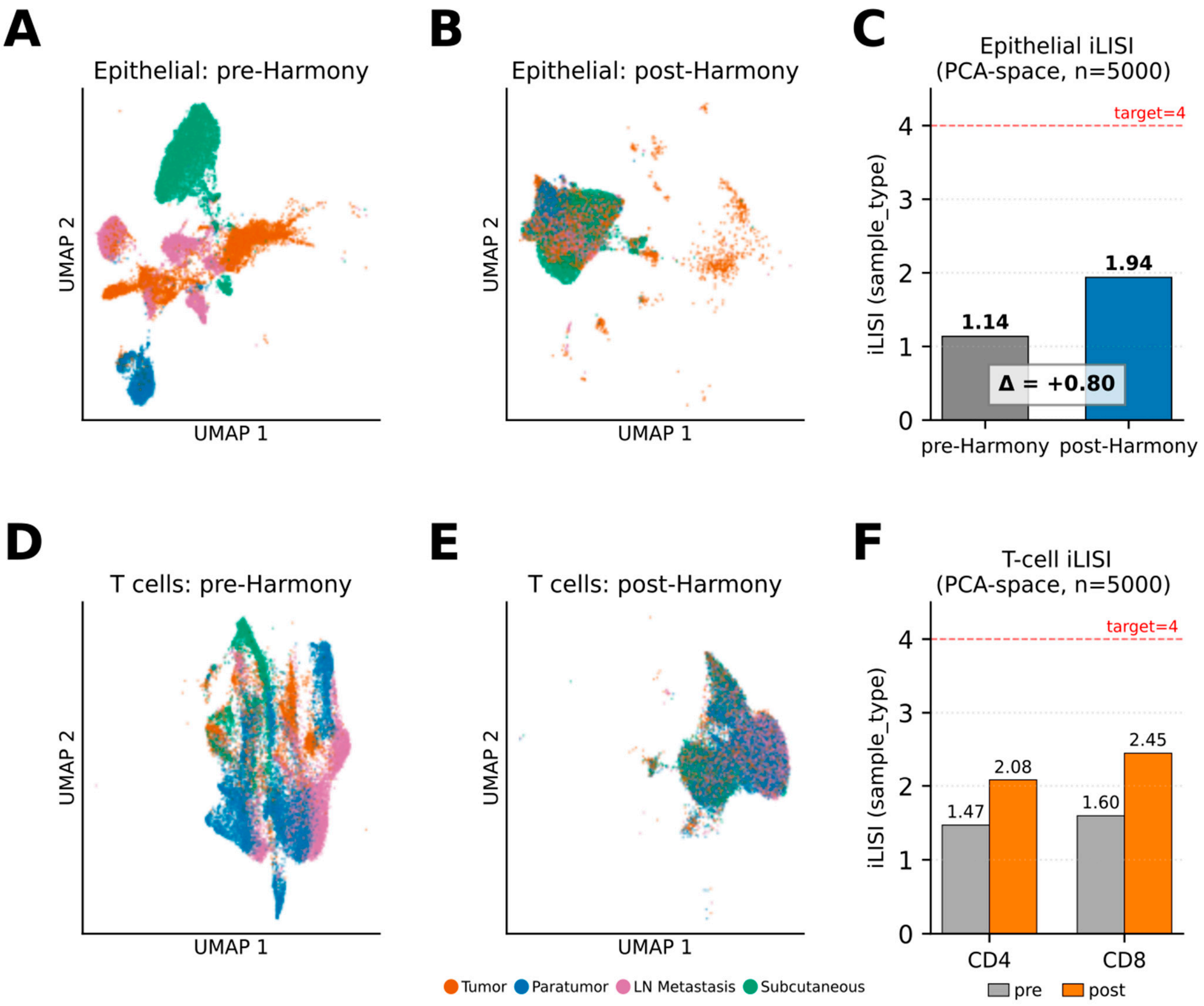

Supplementary Figure S9. Effect of Harmony integration on sample mixing by cell type. UMAPs and iLISI bar plots showing improved sample mixing for epithelial cells and T cells after Harmony correction.

Supplementary Tables

Supplementary Table S1. Gene set composition, pre- vs. post-Harmony robustness, and CD4/CD8 lineage stratification of T-cell functional state signatures in the PTC single-cell atlas.

Supplementary Table S2. CellChat permutation testing of 13 manually curated checkpoint ligand–receptor pairs in GSE184362 tumor and lymph node metastasis samples.

Supplementary Table S3. Comparison of CREM correlation with internally derived TCR-IEG panel versus formal cAMP-related gene signatures in the PTC T-cell atlas.

Supplementary Table S4. Spearman correlations between CREM expression and proliferation- and EMT-associated MSigDB Hallmark pathways in bulk TCGA–THCA (n = 505).

Supplementary Table S5. Stratified Cox proportional hazards analysis of CREM_z and progression-free interval (PFI) according to ImmuneScore and tumor purity in TCGA–THCA (n = 505).

Supplementary Table S6. Spearman correlations between CREM expression and 19 immune-cell features in bulk TCGA–THCA (n = 505) across three deconvolution methods.

Supplementary Table S7. Partial Spearman correlation between CREM and HALLMARK_E2F_TARGETS after graded adjustment for immune-cell covariates in TCGA–THCA (n = 505).

Supplementary Table S8. Multivariate Cox proportional hazards models for progression-free interval (PFI) comparing baseline (4-covariate) versus immune-adjusted (9-covariate) specifications in TCGA–THCA (n = 505, 52 events).

Supplementary Table S9. Sensitivity analyses of multivariate Cox proportional hazards models for CREM expression and progression-free interval (PFI) under alternative covariate specifications in TCGA–THCA (n = 504, 52 events).

Supplementary Table S10. Global and covariate-specific tests of the proportional hazards assumption for the Cox models listed in Supplementary Table S9.

Supplementary Table S11. BRAF V600E and RAS genotype status across the 11 patients of the GSE184362 single-cell PTC cohort.

Supplementary Table S12. CellChat-inferred ANXA1–EGFR interaction probabilities (lr_means) and permutation-test significance (P, FDR) from different source cell types to epithelial cells in tumor and lymph node metastasis samples (1000 permutations).

Supplementary Table S13. Concordance between manual lineage annotation and SingleR (Human Primary Cell Atlas reference) across major lineages in the Harmony-integrated PTC atlas (n = 54,045 cells).

Supplementary Table S14. Differential CellChat analysis of ANXA1–EGFR interaction strength (lr_means) comparing CREM-high versus CREM-low T cells as senders to epithelial cells across five evaluable samples (paired Wilcoxon p = 0.63).
