# Supplementary material for: CREM Marks TCR-Driven T-Cell Activation and Associates with Favorable Prognosis in Papillary Thyroid Carcinoma: A Single-Cell and Bulk Transcriptomic Study
Source: Int J Mol Sci. 2026 Aug 18;27(16):7387. doi: 10.3390/ijms27167387 (PMC13513026; doi:10.3390/ijms27167387)
Supplement: Supplementary file 1 [file ijms-27-07387-s001.zip › ijms-4463250-supplementary/Supplementary File(s)/Supplementary_Methods .pdf]

Supplementary Methods

S1. Data sources and preprocessing

Single-cell RNA-seq. The discovery atlas was obtained from GEO accession GSE184362 (23 samples from 11 patients with papillary thyroid carcinoma across four tissue contexts: primary tumor, paratumor, lymph-node metastasis, and subcutaneous metastasis). The independent validation cohort was obtained from GEO accession GSE193581; only the 7 PTC tumor samples (of 10 anaplastic thyroid carcinoma, 7 PTC, and 6 adjacent normal thyroid tissues) were used for external validation of the CREM-associated T-cell transcriptional program.

Bulk data. Bulk transcriptomic (HTSeq counts/TPM), clinical, and DNA methylation data for TCGA-THCA were retrieved from the Genomic Data Commons (GDC) portal. Progression-free interval (PFI) endpoints were curated from the PanCanAtlas TCGA-CDR resource. Reference gene sets were obtained from the Molecular Signatures Database (MSigDB) v2024.1 (Hallmark collection).

S2. Single-cell quality control, integration, and annotation

Quality control retained cells with >=200 detected genes, <7500 genes per cell (upper-bound doublet proxy), <20% mitochondrial transcripts, and >=500 total counts; genes detected in >=3 cells were retained. Counts were library-size normalized and log1p-transformed, followed by highly-variable-gene selection and principal component analysis. Batch effects across samples were corrected with Harmony using the sample as the batch key. Unsupervised clustering used the Leiden algorithm at resolution 1.0 for both the full atlas and the T-cell sub-atlas.

Doublet control was applied at two levels: an upper-bound gene-count filter (<7500 genes) as an initial doublet proxy, and cluster-level resolution during manual lineage annotation, in which clusters showing balanced co-expression of canonical markers from distinct lineages were assigned to their dominant lineage by marker score; one small cluster (236 cells) without a dominant lineage was removed as doublet-enriched. The final atlas comprised 37 clusters, all 100% lineage-pure. Of the 55,015 post-QC cells, 54,045 were assigned to the seven major lineages (the remaining 970 cells were 860 Plasma cells and 110 Mast cells, which fall outside the seven-lineage scheme). Manual annotation was cross-validated against SingleR (Human Primary Cell Atlas reference) on these 54,045 seven-lineage cells, yielding 92.8% concordance (Supplementary Table S13).

S3. T-cell functional-state scoring

CD4+ and CD8+ T cells were isolated to construct a lineage-specific sub-atlas with recomputed dimensionality reduction. Six canonical T-cell functional states and a post hoc TCR-IEG-activated (cAMP_Activated) state were scored with scanpy.tl.score_genes using the gene panels in Supplementary Table S1: Naive (CCR7, SELL, TCF7, LEF1, IL7R); Exhausted (PDCD1, HAVCR2, LAG3, TIGIT, TOX, CTLA4); Effector/cytotoxic (GZMA, GZMB, GZMK, GZMH,

PRF1, IFNG, GNLY, NKG7); Effector Memory (GZMK, EOMES, CXCR3, CD27, CXCR4); Tfh (CXCR5, BCL6, ICOS, PDCD1, CXCL13); Th17 (IL17A, IL17F, RORC, CCR6, IL23R); and the six-gene TCR-IEG-Activated signature (RGCC, JUNB, CXCR4, NR4A1, NR4A2, ZFP36). FOXP3+ regulatory T cells were identified using a canonical five-gene Treg panel (FOXP3, IL2RA, CTLA4, IKZF2, TIGIT), scored with scanpy.tl.score_genes. Cells were assigned to mutually exclusive functional categories (Exhausted-only, Effector-only, Dual, Other) by median-split classification on the corresponding scores.

S4. Cell-cell communication analysis

Cell-cell communication was inferred on primary tumor and lymph-node metastasis samples using the CellChat model as implemented in liana-py v1.7.3 (li.mt.cellchat), with use_raw = True, expr_prop = 0.05, n_perms = 1000, and seed = 41. A consensus ligand-receptor resource was supplemented with 13 manually curated immune-checkpoint pairs to guarantee they were tested: CD80-CD28, CD80-CTLA4, CD86-CD28, CD86-CTLA4, LGALS3-LAG3, LGALS9-HAVCR2, NECTIN2-CD226, NECTIN2-TIGIT, and TIGIT-NECTIN3 (9 tested pairs), plus PVR-TIGIT, PVR-CD226, CD274-PDCD1, and PDCD1LG2-PDCD1 (4 pairs filtered by the 5% expression floor and therefore not tested). Population-level significant interactions were identified after multiple-testing correction. The per-pair results are reported in Supplementary Table S2; the ANXA1-EGFR permutation test by source compartment is reported in Supplementary Table S12; and the per-sample CREM-high versus CREM-low differential-communication analysis is reported in Supplementary Table S14.

S5. Survival analysis and pathway scoring

Cox proportional-hazards models (lifelines v0.30.0, CoxPHFitter) were used to evaluate the association between CREM expression and PFI, adjusted for age, sex, AJCC stage, and BRAF V600E status. The primary model used CREM dichotomized at the median (CREM_high) on the complete-case cohort with PFI.time > 0 (n = 504, 52 PFI events); continuous z-scored CREM (CREM_z) models on the full primary-tumor cohort (n = 505) were used for the stratified and immune-adjusted sensitivity analyses (Supplementary Tables S5 and S8). Because AJCC 8th-edition staging uses age 55 as a boundary, age-stratified models (<55 vs. >=55 y), a CREM x age-group interaction term, a sex-adjusted model, a stage-free sensitivity model, and a continuous BRAF-RAS score (BRS) model were fitted (Supplementary Table S9). The proportional-hazards assumption was evaluated with Schoenfeld residuals (Supplementary Table S10). Hallmark pathway scores (MSigDB v2024.1) were computed as mean z-scores of log2(TPM+1).

S6. DNA methylation and bulk immune deconvolution

DNA methylation at the CREM locus was quantified from the TCGA-THCA 450K methylation matrix (beta values). Spearman correlations between CREM-locus CpG beta values and CREM mRNA expression were computed across tumors (42 CpG sites), and tumor-versus-normal methylation was compared at those sites. Bulk immune deconvolution was performed with MCP-counter (10 cell populations) and ESTIMATE (ImmuneScore, ESTIMATEScore, StromalScore),

supplemented by six ssGSEA immune signatures (Macrophage, MDSC, Regulatory_T_cell, Activated_CD8_T_cell, Type_1_T_helper_cell, Activated_CD4_T_cell); correlations with CREM are reported in Supplementary Table S6, partial Spearman analyses in Supplementary Table S7, and immune-adjusted Cox models in Supplementary Table S8.

S7. External validation (GSE193581)

The CREM-IEG association was validated in the 7 PTC tumors of GSE193581. T cells were subset using the series' cell-type annotation; counts were library-size normalized (CPM) and log1p-transformed, and Spearman correlation of CREM against the six-gene IEG signature (RGCC, JUNB, CXCR4, NR4A1, NR4A2, ZFP36) was computed at the single-cell level (n = 5,454 T cells) and at the patient level (n = 7).

S8. Statistical analysis, reproducibility, and software

Spearman's rank correlation, Fisher's exact test, the Cochran-Mantel-Haenszel test, and the Wilcoxon rank-sum test with Benjamini-Hochberg correction were applied as appropriate. All analyses used fixed random seeds. Software versions: Python 3.11; scanpy 1.11.5, liana 1.7.3, lifelines 0.30.0, pandas 2.3.3, numpy 1.26.4, scipy 1.17.1, statsmodels 0.14.6; R packages Harmony, Leiden, SingleR (Human Primary Cell Atlas reference), MCP-counter, and ESTIMATE. The complete analysis code is openly available at https://github.com/linghao1992-sketch/crem-ptc.

S9. Cox model sensitivity analyses (Primary + M0-M6)

To assess the robustness of the CREM-PFI association, a series of Cox proportional-hazards sensitivity models were fitted on the complete-case cohort (n = 504, 52 PFI events): the Primary model (CREM + age + stage + BRAF V600E); M0 (CREM + age + stage); a sex-adjusted model (CREM + age + sex + stage); age-stratified models M2a (age <55 y) and M2b (age >=55 y) reflecting the AJCC 8th-edition age boundary; M3 (CREM x age-55 interaction); M4 (stage-free: age + sex + BRAF); and M6 (Primary + continuous BRAF-RAS score, BRS). For each model the hazard ratio, 95% confidence interval, Wald P value, sample size, number of events, and events-per-variable (EPV) are reported (Supplementary Table S9).

S10. Proportional-hazards assumption testing

The proportional-hazards assumption for each Cox model (M0-M6) was evaluated using Schoenfeld residuals. The global chi-square statistic and P value, and the minimum covariate-level P value (with the corresponding term), are reported for each model (Supplementary Table S10); all global tests were non-significant (P > 0.05), supporting the proportional-hazards assumption.

S11. Patient genotyping (BRAF / RAS)

BRAF V600E and RAS (N/H/K) mutation status for the 11 patients of the discovery cohort were determined from the corresponding genomic data. Seven patients were BRAF V600E mutant and four were BRAF wild-type; all 11 patients were RAS wild-type. Patient-level genotype assignments are listed in Supplementary Table S11.

S12. ANXA1-EGFR permutation test by source compartment

The cell-type specificity of the ANXA1-EGFR ligand-receptor axis was assessed by computing the CellChat-inferred interaction strength (lr_means) from each source cell compartment to epithelial cells, with permutation testing (n_perms = 1000) and Benjamini-Hochberg FDR correction. The lr_means, permutation P value, FDR, and significance call (FDR < 0.05) for each source compartment are reported in Supplementary Table S12.

S13. SingleR versus manual annotation concordance

Manual lineage annotation of the single-cell atlas was cross-validated against automated annotation with SingleR using the Human Primary Cell Atlas reference. Concordance was computed per lineage and overall across the 54,045 cells assigned to the seven major lineages (i.e., excluding the 860 Plasma cells and 110 Mast cells from the 55,015-cell post-QC atlas; overall concordance 92.8%; Supplementary Table S13).

S14. CREM-high versus CREM-low differential communication

Differential ANXA1-EGFR communication from CREM-high versus CREM-low T cells to epithelial cells was quantified per sample. For each evaluable sample, T cells were split by median CREM expression into CREM-high and CREM-low subsets, and the CellChat-inferred ANXA1-EGFR interaction strength (lr_means) to epithelial cells was computed for each subset. Five samples were evaluable (PTC10_RightLN, PTC11_RightLN, PTC7_RightLN, PTC8_T, PTC9_T); the paired difference was tested with a two-sided paired Wilcoxon signed-rank test (mean lr_means 0.845 CREM-high vs. 0.816 CREM-low; p = 0.63; Supplementary Table S14).
